# Global analysis of the eukaryotic pathways and networks regulated by *Salmonella typhimurium *in mouse intestinal infection *in vivo*

**DOI:** 10.1186/1471-2164-11-722

**Published:** 2010-12-20

**Authors:** Xingyin Liu, Rong Lu, Yinglin Xia, Jun Sun

**Affiliations:** 1 Department of Medicine, Gastroenterology & Hepatology Division, University of Rochester, 601 Elmwood Avenue, Rochester, NY 14642, USA; 2 Department of Biostatistics and Computational Biology, University of Rochester, 601 Elmwood Avenue, Rochester, NY 14642, USA; 3 Department of Microbiology and Immunology, University of Rochester, 601 Elmwood Avenue, Rochester, NY 14642, USA; 4 Wilmot Cancer Center, University of Rochester, 601 Elmwood Avenue, Rochester, NY 14642, USA

## Abstract

**Background:**

Acute enteritis caused by *Salmonella *is a public health concern. *Salmonella *infection is also known to increase the risk of inflammatory bowel diseases and cancer. Therefore, it is important to understand how *Salmonella *works in targeting eukaryotic pathways in intestinal infection. However, the global physiological function of *Salmonella *typhimurium in intestinal mucosa *in vivo *is unclear. In this study, a whole genome approach combined with bioinformatics assays was used to investigate the *in vivo *genetic responses of the mouse colon to *Salmonella*. We focused on the intestinal responses in the early stage (8 hours) and late stage (4 days) after *Salmonella *infection.

**Results:**

Of the 28,000 genes represented on the array, our analysis of mRNA expression in mouse colon mucosa showed that a total of 856 genes were expressed differentially at 8 hours post-infection. At 4 days post-infection, a total of 7558 genes were expressed differentially. 23 differentially expressed genes from the microarray data was further examined by real-time PCR. Ingenuity Pathways Analysis identified that the most significant pathway associated with the differentially expressed genes in 8 hours post-infection is oxidative phosphorylation, which targets the mitochondria. At the late stage of infection, a series of pathways associated with immune and inflammatory response, proliferation, and apoptosis were identified, whereas the oxidative phosphorylation was shut off. Histology analysis confirmed the biological role of *Salmonella*, which induced a physiological state of inflammation and proliferation in the colon mucosa through the regulation of multiple signaling pathways. Most of the metabolism-related pathways were targeted by down-regulated genes, and a general repression process of metabolic pathways was observed. Network analysis supported IFN-γ and TNF-α function as mediators of the immune/inflammatory response for host defense against pathogen.

**Conclusion:**

Our study provides novel genome-wide transcriptional profiling data on the mouse colon mucosa's response to the *Salmonella typhimurium *infection. Building the pathways and networks of interactions between these genes help us to understand the complex interplay in the mice colon during *Salmonella *infection, and further provide new insights into the molecular cascade, which is mobilized to combat *Salmonella*-associated colon infection *in vivo*.

## Background

*Salmonella *is a leading cause of gastrointestinal disease worldwide [1]. The overall estimated 2-4 million cases of *Salmonella*-induced gastroenteritis constitute a significant economic loss of productive work time, reported to exceed $2 billion annually [2-4]. *Salmonella *infection can appear as enteric fever, gastroenteritis, bacteremia, or extra intestinal focal infection. Central to *S*. typhimurium pathogenesis is its ability to induce intestinal inflammation [5]. Beyond the public health problem caused by *Salmonella*, recent studies further have demonstrated that *Salmonella *infection increases the risk of developing inflammatory bowel diseases (IBDs) [6]. Moreover, *Salmonella *infection increases the risk of other gastrointestinal (GI) diseases, including chronic inflammation and gallbladder cancer [6,7]. Therefore, it is important to understand which pathway *Salmonella *target that may potentially contribute to chronic inflammation and tumorigenesis.

Increasing evidence links some *Salmonella *species to carcinogenesis, whereas others appear promising in the diagnosis, prevention, or treatment of cancers [8]. *Salmonella *and its derivatives prefer solid tumors over normal tissue in animal models [9,10]. Using *Salmonella *DNA or plasmids to cancer therapy is a very active field. Alive, mutated, non-invasive *Salmonella *has been used as a vector to specifically target cancer cells [11]. It may be controversial if *Salmonella *could contribute to intestinal inflammation and cancer. Hence, it is necessary to understand the global facets of *Salmonella *in the intestine using animal models.

Gene expression array technology is a powerful tool in expanding the understanding of host-pathogen interactions. Although many genes that respond to *Salmonella *infection have been identified in previous genomics research [12-15], the majority of such studies generally result in the identification of hundreds of genes that are involved in many different biological processes and pathways. The mouse model is widely used to study the mechanisms of systemic salmonellosis [16,17]. A number of reports have described host transcriptional responses to bacterial infection using microarrays [5,18-20]. The intestinal epithelial cells are constitutively exposed to commensal flora and pathogenic bacteria, and they play barrier, structural, and host defense roles [21-31]. The global physiological function and pathway analysis of *Salmonella *on intestinal mouse mucosa is unclear. We lack the knowledge of the most affected gene networks and pathways in response to *Salmonella *infection in mouse colon mucosa *in vivo*.

In this study, we focused on the intestinal responses at the early phase (8 hours) and the late phase (4 days) after *Salmonella *infection. The histologic assay of intestine indicated that 8 hours is the early stage of the *Salmonella *infection and 4 days is the late stage of infection [17]. Hence, we chose these two time courses in the current study. We used the *Salmonella *typhimurium wild-type SL1344 because it is a mouse-virulent strain and well-documented in *in vitro *and *in vi*vo studies [17]. A whole genome approach combined with bioinformatics assays was used to dissect the genetic responses of the mouse colon to *Salmonella in vivo*.

## Methods

### Bacterial strains and growth condition

*Salmonella *typhimurium wild-type strain SL1344 (WT) was used in this study (provided by Dr. Jorge Galan at the Yale University) [32]. Non-agitated microaerophilic bacterial cultures were prepared by inoculating 10 ml of Luria-Bertani broth with 0.01 ml of a stationary phase culture followed by overnight incubation (~18 h) at 37°C as previously described [33].

### Streptomycin pre-treated mouse model

Animal experiments were performed using specific-pathogen-free female C57BL/6 mice (Taconic, Hudson, NY, USA) that were 6-7 weeks old. The protocol was approved by the University of Rochester University Committee on Animal Resources (UCAR). Water and food were withdrawn 4 hours before oral gavage with 7.5 mg/mouse of streptomycin. Afterwards, animals were supplied with water and food ad libitum. Twenty hours after streptomycin treatment, water and food were withdrawn again for 4 hours before the mice were infected with 1 × 10^7 ^CFU of *S. Typhimurium *(100 μl suspension in HBSS) or treated with sterile HBSS (control) by oral gavage as previously described [34]. At 8 hours and 4 days after infection, mice were sacrificed and tissue samples from the intestinal tracts were removed for analysis, as previously described [34].

### Sample RNA preparation

Mice were sacrificed at 8 hours and 4 days after *Salmonella *infection, and tissue samples from the intestinal colon mucosa were removed. Total RNAs were isolated using TRIzol reagent (Invitrogen, Carlsbad, CA, USA) following the manufacturer's protocol, followed by on-column digestion of DNA using the RNeasy Mini Kit (Qiagen, Valencia, CA, USA). RNA quantity and quality were assessed with a Beckman Coulter DU 640 Spectrophotometer (Beckman Coulter, Brea, CA, USA) and Agilent 2100 Bioanalyzer (Agilent, Santa Clara, CA, USA), following the manufacturer's protocols.

### Gene array processing and statistical analysis

The biotinylated single-stranded cDNA was prepared from 100 ng total intact RNA extracted from uninfected mouse control samples. Mouse mucosa at 8 hours and 4 days post infection was collected. Mouse cDNA was hybridized to the Mouse Gene 1.0 ST array, a microarray chip containing 28,000 sequenced mouse genes (Affymetrix). After hybridization, the array was washed and stained with streptavidin-phycoerythrin, and scanned in a proprietary Affymetrix scanner, according to the GeneChip^® ^Whole Transcript Sense Target Labeling Assay manual. The fluorescence values for each feature on the array were measured and recorded. Command Console software (Affymetrix) was used to produce a CEL file. All procedures were performed in three biological replicates at the Functional Genome Center of the University of Rochester. The data were processed with Expression Console (Affymetrix) using the PLIER algorithm *(Affymetrix *Guide to Probe Logarithmic Intensity Error (PLIER) Estimation. http://www.affymetrix.com/support/technical/technotes/plier_technote.pdf), which uses quantile normalization. Fold change was calculated for each strain relative to the uninfected control. Statistical significance (p value) was calculated by Student's *t *test, based on the results of three arrays per condition. Insignificant genes that changed by less than 1.2 fold and p value > 0.05 were removed from subsequent analysis. We set 1.2 as the cut-off (p < 0.05) standard in order to analyze more genes involved in intestinal homeostasis and this cut-off is acceptable in the field [35,36]. The false discovery rate (Q value) was calculated for each *P*-value using R program according to the Storey and Tibshirani method [37]. We also estimated false discovery rate using Significance Analysis of Microarrays [38]. The microarray data used in this analysis have been submitted to NCBI GEO database under accession number GSE22215.

### Functional interpretation of microarray data as well as pathway and network analysis

Ingenuity Pathways Analysis (IPA) (Ingenuity Systems http://www.ingenuity.com) is a web-based software application tool which is designed to organize biological information in a way that allows one to gain a high level overview of the general biology that is associated with microarray data [39-41].

In this study, the biofunctional analysis identified the molecular and cellular function that was most significant to the data set as a whole, thus generating functional interpretation of microarray data. Fischer's exact test was used to calculate a p-value determining the probability that each biofunction assigned to that data set is due to chance alone. IPA Canonical Pathways Analysis tool was used to identify the signaling and metabolic pathways associated with the database. Genes from the dataset that met the fold change cut-off of 1.2 were considered for the analysis. The significance of the association between the dataset and the canonical pathway was measured in two ways, the ratio and the significance. The ingenuity network analysis was used to display an interactive graphical representation of the interrelationships between molecules.

### Real-time quantitative reverse transcriptase PCR

Quantitative PCR technology was used to verify the differential expression of 23 genes, including some genes around the two networks IFN-γ and TNF-α, at early and late response stages as identified by the microarray. Total RNA was reverse transcribed with oligoDT primer using an Invitrogen SuperScript III kit. The cDNA was subject to qRT-PCR using SYBR Green Supermix (Bio-Rad, Hercules, CA, USA). Primers of target genes are listed in Additional file 1 Table S1. The amplification conditions were optimized for the MJ research DNA Engine instrument, using melting curve and electrophoresis analysis. The threshold cycle (Ct) was determined, i.e., the cycle number at which the fluorescence of the amplified product crosses a specific threshold value in the exponential phase of amplification. Relative quantification of target gene expression was evaluated using the comparative cycle threshold (*C*_T_) method as previously described by Livak and Schmittgen [42]. A value of p ≤ 0.05 was considered statistically significant. Correlation analysis was performed by comparing expression ratios from the microarray results with the ratios tested by the qRT-PCR analysis. The Pearson correction coefficient between the qRT-PCR and microarray was analyzed.

### *Salmonella*-induced mouse cytokine secretion

Mouse blood samples were collected by cardiac puncture and placed in tubes containing EDTA (10 mg/ml) [43,44]. Mouse cytokines were measured using mouse cytokine 10-Plex Panel kit (Invitrogen) according to the manufacturer's instructions. Briefly, beads of defined spectral properties were conjugated to protein-specific capture antibodies, and then samples were added (including standards of known protein concentration, control samples, and test samples) into the wells of a filter-bottom microplate where proteins bound to the capture antibodies over the course of a 2 hour incubation. After washing the beads, protein-specific biotinylated detector antibodies were added and incubated with the beads for 1 hour. After removal of excess biotinylated detector antibodies, streptavidin conjugated to the fluorescent protein, then R-Phycoerythrin (Streptavidin- RPE) was added and allowed to incubate for 30 minutes. After washing to remove unbound Streptavidin-RPE, the beads were analyzed with the Luminex detection system (PerkinElmer CS1000 Autoplex Analyzer, Covina, CA, USA) [45].

### Immunoblotting

Mouse colonic mucosa was collected by scraping the mouse colon, including proximal and distal regions [46]. Cells were sonicated in lysis buffer (1% Triton X-100, 150 mM NaCl, 10 mM Tris pH 7.4, 1 mM EDTA, 1 mM EGTA pH 8.0, 0.2 9 mM sodium ortho-vanadate, protease inhibitor cocktail). The protein concentration was measured using BioRad Reagent (BioRad). Cultured cells were rinsed twice in ice-cold HBSS, lysed in protein loading buffer (50 mM Tris, pH 6.8, 100 mM dithiothreitol, 2% SDS, 0.1% bromophenol blue, 10% glycerol), and sonicated. Equal amounts of protein were separated by SDS-polyacrylamide gel electrophoresis, transferred to nitrocellulose, and immunoblotted with primary antibodies. The following antibodies were used: monoclonal Rabbit anti-Akt (Cell Signal, Beverly, MA, USA), Anti-Villin (Santa Cruz Biotechnology, Santa Cruz, CA, USA) and anti-actin (Sigma-Aldrich, Milwaukee, WI, USA).

### Histology and immunofluorescence of mouse colon

Colonic tissues from the proximal and distal portion of the colon were freshly isolated and embedded in paraffin wax after fixation with 10% neutral 10 buffered formalin. Sections (5 μm) were stained with hematoxylin and eosin (H and E). For immunofluorescence microscopy, tissue samples were processed for immunofluorescence as described previously [43,44,47-49]. In short, tissue samples were blocked in PBS containing 5% BSA and 10% goat serum, then incubated overnight with Sheep anti-Brdu (treated sample using HCL for 90 min before incubating) or Rrabbit anti-Akt antibodies. Immunofluorescence staining was performed by utilizing red fluorescent anti-sheep or anti-rabbit IgG (Alex 594, Invitrogen) for one hour, then cover slips were washed three times with PBS (10 min/each time). DAPI was added in the final wash, then mounted with SlowFade (SlowFade^® ^AntiFade Kit, Molecular Probes, Invitrogen) and followed by a cover slip. Edges were sealed to prevent drying. Samples were viewed with an Olympus Ax70 microscope. Captured images were analyzed using Image-pro plus 5.0 software.

## Results

### Global gene differential expression during the time course of *Salmonella *infection in the mouse colon mucosa

In this current study, we focused on the mouse colon mucosa's responses to *Salmonella *infection at both 8 hours and 4 days post infection *in vivo*. Previous studies indicate that the 8 hours post-infection provides insights into the early events of *Salmonella *infection in the colon, whereas investigation of the gene expression response at 4 days post-infection can show the outcome of infection and inflammation [43,44,47,48]. Hence, we selected genes that changed in response to *Salmonella *infection at 8 hours and 4 days. In total, we hybridized 9 different samples (3 individual mice from each of the three groups) to the microarray chip in this study.

FDR and nominal P were similar at 4 days, the analysis was based on the nominal P values (all FDR values < 10% when nominal P < 0.05). At 8 hours, the FDR values were high for all probe sets (> 46%). However, we included this time point because it allows us to investigate how many genes began to respond at the early stage of infection and how many genes are dependent on the time-course responses.

### Top differentially regulated genes in response to infection

**Figure 1:**
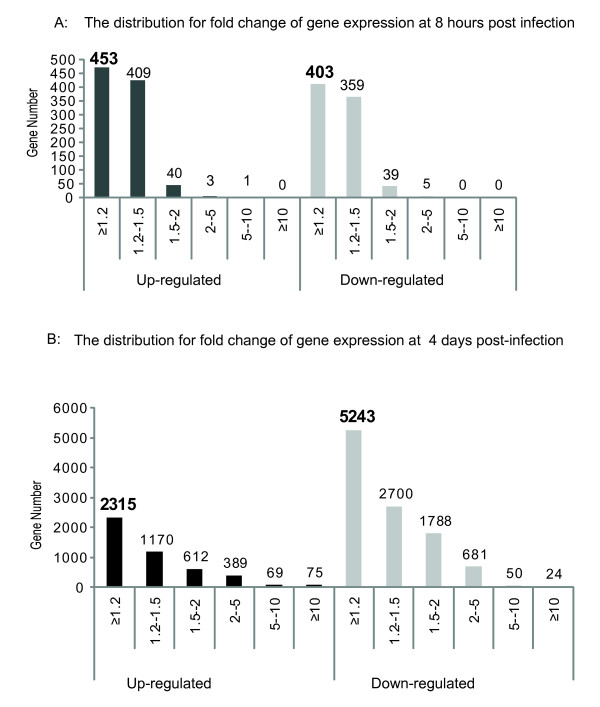
**The distribution of gene expression regulated by *Salmonella typhimurium *in mouse intestinal infection *in vivo***. A. The distribution for fold change of gene expression 8 hours post infection with *Salmonella*. B. The distribution for fold change of gene expression 4 days post-infection with *Salmonella*.

Differential expression analyses between the normal control and the *Salmonella*-infected group were carried out with GeneSifter software (VizX Labs, Seattle, WA). GeneSifter™ is unique in the field of microarray data analysis software because it is the only package that is wholly web-based http://www.geospiza.com/Products/AnalysisEdition.shtml. Of the 28,000 genes represented on the array, our analysis of mRNA expression in mouse colon mucosa showed that a total of 856 genes were expressed differentially (Figure 1A and Additional file 2 Table S2) in the pathogenic SL1344 infected group at 8 hours. Of these 856 genes, 453 genes (53%) were up-regulated and 403 (47%) were down-regulated. 99% selected genes showed 1.2-2 fold times change (Figure 1A, black and white). There were only 4 up-regulated and 5 down-regulated genes with a fold change range from 2 to 5 times.

At 4 days post-infection, we identified 7558 genes that were expressed differentially (Additional file 3 Table S3). The majority of the selected genes showed moderate alterations in expression of between 1.2 and 2.0 fold (Figure 1B). Figure 1B also showed that 389 up-regulated genes and 681 down-regulated genes with a fold change range from 2 times to 5 times, 69 up-regulated genes and 50 down-regulated genes with a fold change range from 5 times to 10 times, and 75 up-regulated genes and 24 down-regulated genes with a fold change greater than 10 times. The data for differentially expressed genes are shown in Table S2-S3.

For the differential expression genes between animals infected for 8 hours and 4 days, we identified 7127 genes that were expressed differentially during two time course (Additional file 4 Table S4). Total 4951 genes (69% genes) showed down-regulated and 2176 genes (31%) showed up-regulated. 3798 down-regulated genes between animals infected for 8 hours and 4 days showed the similar expression pattern as the gene list between the control and 4 days post-infection group. Therefore, we focused on the differential expression between non infected and infected mice at either 8 hours or 4 days post-infection.

Two genes, *Hist1hle *and *Arnt1 *showed up-regulated between 4 days post-infection and the control group without treatment, but showed down-regulated between 4 days post-infection and 8 hours post-infection. The up-regulated 1328 genes between 4 days and 8 hours post-infection showed the similar expression pattern as the genes between 4 days post-infection to control. Other 846 up-regulated genes were new responses between 4 days and 8 hours post-infection, and 90% of these genes had a fold change range from 1.2 times to 2 times. Three genes, Olfr1257 and other two unknown genes, were down-regulated between 4 days post-infection and control, but up-regulated between 4 days post-infection and 8 hours post-infection. Overall, the data indicate that pathogenic *Salmonella *regulates the gene expression in intestinal mucosa at the early stage of infection, but more profound changes in gene expression occurred in the late stage of infection.

We further analyzed the top up-regulated and down-regulated genes in every infection group to identify the specific responses induced by *Salmonella*-infection (Table 1). Of the 10 annotated up-regulated genes in SL1344 infection at 8 hours, R*ETNLB *as a colon-specific gene has been reported to associate with bacteria infection, and *RETNLB *in situ hybridization occurs in proliferative mouse epithelial cells [50]. IPA data base showed *RETNLB *and *FDPs *were associated with endocrine system disorders; *CAPG*, *ACOT9, FDPS*, and *IMPDH2 *were associated with genetics disorder. *CAPG *encodes a member of the gelsolin/villin family of actin-regulatory proteins. By capping the barbed ends of actin filaments, the encoded CAPG protein contributes to the control of actin-based motility in non-muscle cells [51]. *Salmonella *effector proteins promote bacteria internalization by binding to actin and directly modulating actin dynamics [52-54]. CAPG may be involved in regulation of actin microfilament remodeling which is required for *Salmonella *invasion at the early stage of infection. IPA data base showed that *NOV, TIPIN*, and *IMPDH2 *are associated with cellular growth and proliferation. TIPIN has been shown to interact with Replication protein A [55]. It is also associated with the cell cycle and DNA replication [56]. The other 2 up-regulated genes, *2010109I03RIK *and *FBXW12 *are associated with bio-process and molecular function and diseases.

**Table 1:** Top-differentially expressed gene list at 8 hours and 4 days post-infection.

SL1344 infection at 8 hr				SL1344 infection at 4 day			
**Up-regulated**		**Down-regulated**		**Up-regulated**		**Down-regulated**	

**Gene Name**	**Fold**	**Gene Name**	**Fold**	**Gene Name**	**Fold**	**Gene Name**	**Fold**

*RETNLB*	5.2	*TRDN*	-3.4	*CXCL5*	310.8	*CYP2C18*	-72.7
*2010109I03RIK*	2.2	*SGK1*	-3.0	*S100A8*	221.5	*CES1*	-33.1
*NOV*	2.0	*PLK3*	-2.4	*LRG1*	217.5	*CES3*	-28.6
*SERPINB12*	1.7	*EDN2*	-2.1	*LCN2*	184.1	*NOV*	-27.1
*CAPG*	1.7	*SLC37A2*	-2.0	*S100A9*	144.7	*B3GNT6*	-24.3
*IMPDH2*	1.7	*EMX2*	-2.0	*LTF*	117.7	*SSTR1*	-18.5
*TIPIN*	1.7	*TP53INP1*	-1.9	*CCL8*	111.1	*ARHGAP20*	-15.6
*FBXW12*	1.7	*SSTR1*	-1.9	*IIGP1*	93.2	*ODF3B*	-15.0
*FDPS*	1.7	*C1ORF51*	-1.8	*NOS2*	94.0	*SULT1A1*	-13.5
*ACOT9*	1.7	*TGFBI*	-1.8	*GBP4*	84.6	*CYP4B1*	-13.5

Of the top 10 annotated down-regulated genes in SL1344 infected group at 8 hours, we found most of the genes were relative to the cell cycle and cell death process: *TP53INP1, SGK1*, *SSTR1 *and *EMX2 *were involved in cell cycle; *TP53INP1*, *TGFBI*, *SGK1 *and *PLK3 *were involved in cell death. The result supports that *Salmonella *infection plays a role in regulating the host's cell cycle and cell death process at early stages of infection, as we reported in a recent study [57].

At 4 days post-infection, of the 10 annotated up-regulated genes, 70% genes are involved in inflammatory response and bacterial infection; only *LGR1 *is involved in connective tissue development. Interestingly, *S100 *family proteins are involved in the regulation of a number of cellular processes, and two members of this gene family, *S100A8 *and *S100 A9*, were significantly up-regulated with a large fold change at 4 days post infection relative to non-infected mouse. In agreement with our results, Rodenburg *et al *also reported up-regulation of *S100A8 *and *S100A9 *in the rat colonic mucosa infected by *Salmonella *[58].

Of the top 10 annotated down-regulated genes in SL1344 infected at 4 days (Table 1), 80% genes were involved in the regulation of cell cycle, cellular growth and proliferation and metabolism process, but *SSTR1 *was also involved in inflammatory response. Interestingly, *SSTR1 *showed continual down-regulation at both 8 hours and 4 days post-infection. The *SSTR1 *gene encodes a protein called somatostatin receptor type 1. Somatostatin plays an important role in many physiological processes, such as growth hormone release, cell anti-proliferation, and inhibition of gastrointestinal motility and regulate a variety of signal transduction pathways [59-61]. Moreover, we found that there are three protein families in this list that are involved in xenobiotic metabolism, such as cytochrome P450 (CYP4B1 and CYP2C18), sulfotransferase (SULT1A1), and carboxylesterase (CES1 and CES3). Downregulation of Cytochromes P450 (CYPs) in intestinal epithelium was also shown in response to chicken with *Salmonella *infection [62] and proinflammatory cytokines [63,64].

### Relevant functions, pathways, and biological networks in intestinal mucosa post *Salmonella *infection

In order to identify the gene function and associated biological process for all of differentially expressed genes, the 453 up-regulated genes and 403 down-regulated genes in the 8 hours post-infection group (Figure 1A), and the 2315 up-regulated genes and 5243 down-regulated genes in the 4 days post-infection group (Figure 1B), were assigned to Ingenuity Pathways Analysis (IPA). In total, 229 up-regulated and 254 down-regulated GeneIDS at the 8 hours post-infection group, and 1427 up-regulated and 3368 down-regulated GeneIDS at the 4 days post-infection group, were eligible to biological process and gene network.

An over-representation of a specific biological process does not indicate whether the process in question is being stimulated or repressed overall. The interaction of *Salmonella *with intestine is a key event in the early phases of infection. Still, the signaling steps taking place during this interaction remain largely unknown. Therefore, we used IPA software to further investigate over- or under-represented pathway responses by *Salmonella *infection. In order to further investigate the global expression response to infection with *Salmonella *and to define how individual up-regulated and down-regulated genes interact to have a coordinated role in specific pathways, we further identified potential networks of response to *Salmonella *infection at the early stage and late stage, respectively.

#### Relevant functions, pathways, and biological networks 8 hours post infection

**Figure 2:**
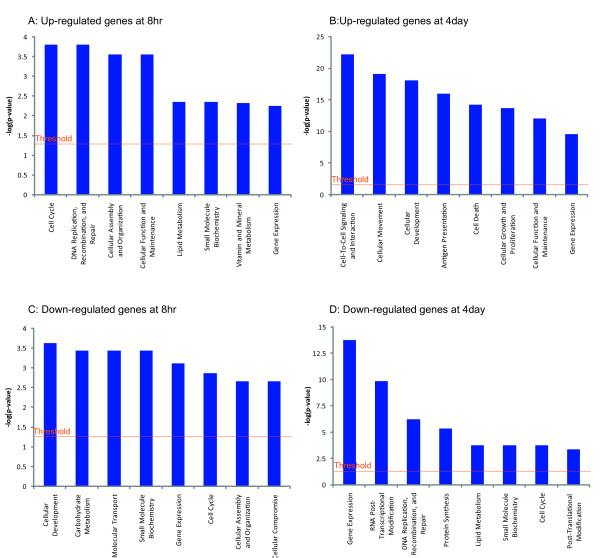
**Functional analysis for a database of differentially expressed genes response to *Salmonella *infection**. A: Functional analysis for up-regulated genes of 8 hours post-infection (Fold times ≥ 1.2 times; P-value ≤ 0.05). B. Functional analysis for up-regulated expressed genes of 4 days post-infection (Fold times ≥ 1.2 times; P-value ≤ 0.05). C: Functional analysis for down-regulated genes of 8 hours post-infection (Fold times ≥ 1.2 times; P-value ≤ 0.05). D: Functional analysis for down-regulated expressed genes of 4 days post-infection.

**Figure 3:**
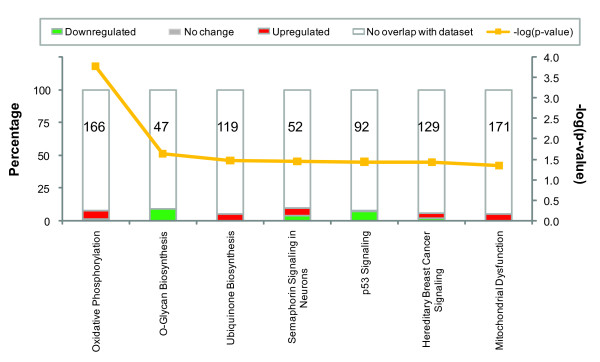
**Canonical pathways identified by IPA associated with differentially expressed genes at 8 hours post-infection**. The 7 most significant canonical pathways across the entire dataset, and across multiple datasets, y-axis displays the significance. For the ratio, taller bars have more genes associated with the canonical pathway than shorter bars. Canonical pathway displays the number of up-regulated (red), down-regulated (green), and unchanged molecules (gray) in each canonical pathway.

In the 8 hours post-infection group, major functional gene categories were especially up-regulated by *Salmonella *infection including DNA replication, recombination and repair, cellular assembly and organization, cellular function and maintenance, and metabolism (Figure 2A). Down-regulated genes at 8 hours post-infection were functionally associated with cellular development and carbohydrate metabolism, molecular transport, and small molecular biochemistry (Figure 2C). However, pathways linked to gene expression and the cell cycle displayed altered regulation (Figure 2A and 2C). The top canonical signaling and metabolic pathways within all of differentially expressed genes at 8 hours post infection group are shown in Figure 3. All the pathways that were affected showed lower significance, which indicated that the pathways linked to these genes had no profound change for cell signaling and transduction at the early stage of infection.

As shown in Figure 3 the most significant pathway associated with differentially expressed genes in 8 hours post-infection is oxidative phosphorylation. The up-regulated genes involved in the pathway were localized in the mitochondria. Accordingly, we found that the pathway related to mitochondrial dysfunction is affected (Figure 3). These data suggested that the signaling response for mitochondrial function is a major event at the early stage of infection. Moreover, we found that most of genes associated with p53 signaling are down-regulated (Figure 3 and Additional file 5 Table S5). Significantly, there was prominent down-regulation mRNA level of *TP53*.

**Figure 4:**
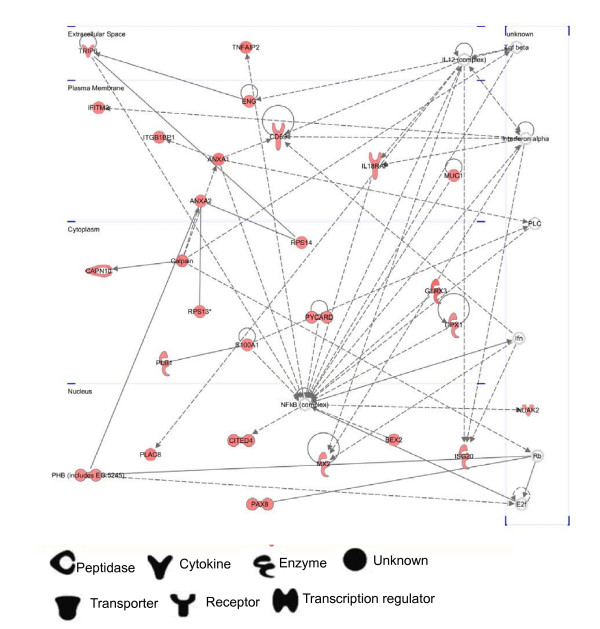
**Ingenuity pathway analysis network 1 depicting relationships among up-regulated genes at the 8 hr post-infection**. Intensity of the red color indicates the degree of up-regulation. Nodes are displayed using various shapes that represent the functional class of the gene product. Edges are displayed with various labels that describe the nature of relationship between the nodes: ___ represents direct relationship; ----- represents indirect relationship; →represents acts on.

We further identify the network at 8 hours post *Salmonella *infection. Network 1 consists of 26 DEGs genes that almost all interact directly with nuclear factor-kappa B transcription factor (NF-κB) (Figure 4 and Additional file 6 Table S6). NF-κB pathway has been previously reported to be activated by *Salmonella *infection [12,65-67]. Our data showed that the network 1 is associated with Cellular Assembly and Organization (*ENG, TRIP6ANXA1, ANXA2ITGB1BP1, CAPN10*), Cellular Function and Maintenance (*ANX1 MUC1*), Antigen Presentation (*ANXA1, ANXA2, IL18RAP CD69*) and Inflammatory Response (*ANXA1, ANXA2, CD69, GPX1, IFITM2, IL18RAP, MX2*).

Overall, we identified 14 highly significant networks of potentially interacting up-regulated genes at 8 hours post-infection group (Additional file 7 Table S7). The highest ranked network is provided as Figure 4. However, for down-regulated genes at 8 hours post infection, we identified 16 highly significant networks of potentially interacting down-regulated genes (Additional file 8 Table S8) and did not identify a network with center regulation.

#### Relevant functions, pathways, and biological networks 4 days post infection

In the 4 days post-infection group (Figure 2B), major functional gene categories that were specifically up-regulated in *Salmonella *infection included cell-to-cell signaling and interaction, cellular movement, cellular development, antigen presentation, cell death, cellular growth and proliferation, and cellular function and maintenance. The pathways associated with RNA post-transcriptional modification, DNA replication, recombination, and repair, protein synthesis, lipid metabolism, post-translational modification and cell cycle were down-regulated (Figure 2D), whereas pathways linked to gene expression displayed altered regulation (Figure 2B and 2D).

We identified canonical pathways involving signaling that is associated with differential genes at 4 days post-infection (Additional file 9 Table S9 and Table 2). Table 2 lists the 20 most significant pathways for fold ≥ 1.2. As shown in Table 2 diverse and complex signals related to cell growth and proliferation, apoptosis signal, and cell immune/inflammatory transduction pathways are involved in the colon mucosa at 4 days post-infection.

**Table 2:** Canonical pathways identified by IPA associated with differentially expressed genes of 4 days post-infection (p ≤ 0.05; fold times ≥ 1.2 times. +: activation; -: inhibition).

Ingenuity signaling Pathways	-log (p-value)	Ratio	Up/down	Effect	Function (From IPA)
NRF2-mediated Oxidative Stress Response	5.25E00	4.37E-01	24/56	+	Cell Death; Developmental Disorder
Antigen Presentation Pathway	3.79E00	4.36E-01	17/0	+	Genetic Disorder; Immunological Disease Gene Expression; Infection Mechanism
Estrogen Receptor Signaling	3.47E00	4.37E-01	4/48	-	Cellular Growth and Proliferation
Xenobiotic Metabolism Signaling	3.57E00	3.60E-01	30/75	-	Lipid Metabol is m; Small Molecule Biochemistry Vitamin and Mineral Metabolism
IL- 9 Signaling	3.4E00	5.41E-01	12/8	+	Cell Death; Cellular Growth and Proliferation
EIF2 Signaling	3.32E00	4.2E-01	10/32	-	Protein Synthesis; Amino Acid Metabolism; Post-Translational Modification
Glucocorticoid Receptor Signaling	2.79E00	3.64E 01	39/63	+	Cellular Development; Gene Expression; Cellular Growth and Proliferation
Regulation of eIF4 and p70S6K Signaling	2.74E00	3.85E-01	11/39	-	Gene Expression; Protein Synthesis and Cancer
Role of PKR in Interferon Induction and Antiviral	2.73E00	5E-01	17/6	+	Cell Death; Cellular Development; Immune Response
DNA Methylation, Transcriptional Repression Signaling	2.51E00	5.65E-01	0/13	-	Gene Expression; DNA Replication, Recombination and Repair; Cell Cycle
IL-4 signaling	2.47E00	4.46E -01	19/14	+	Cellular Development; Hematopoietic
LPS/IL-1 Mediated Inhibition of RXR Function	2.39E00	3.49E-01	27/48	-	Lipid Metabolism;Small Molecule Biochemistry
Retinoic acid Mediated Apoptosis Signaling	2.35E00	4.09E-01	12/6	+	Cell Death; Embryonic Development; Cell Morphology
EGF Signaling	2.35E00	4.69E- 01	9/14	+	Gene Expression; Cell cycle, Cellular Growth and Proliferation
Interferon Signaling	2.29E00	5.33E-01	15/1	+	Organism Injury and Inflammatory Response Cellular Growth and Proliferation
P38 MAPK Signaling	2.21E00	4.23E-01	21/20	+	Gene Expression; Cell Death; Cell Signaling
LPS-stimulated MAPK Signaling	2.2E00	4.23E-01	15/18	+	Cell Death; Gene Expression; Cellular Development.
Insulin Receptor Signaling	2.01E00	3.86E-01	14/40	-	Cancer; Carbohydrate Metabolism; Molecular Transport
Acute Phase Response Signaling	2E00	3.96E-01	10/9	+	Hematological System Development and Function; Hematological Disease; Cell Death
mTOR Signaling	1.99E00	3.88E^-^01	41/28	+	Gene Expression; Protein Synthesis; Cell Morphology
LXR/RXR Activation	1.88E00	4.14E-01	9/20	-	Lipid Metabolism; Molecular Transport; Small Molecule Biochemistry

These data revealed a unique landscape where induction of certain pathways limits the inflammatory response (e.g., IL-4, IL-9 and Interferon signaling), and was coupled with promoting the inflammatory response, such as acute phase response signaling and glucocorticoid receptor signaling. Pathway analysis revealed that two signaling pathways related to protein synthesis (EIF2 signaling as well as Role of PKR in Interferon induction and antiviral response) and three pathways related to lipid metabolism (Xenobiotic metabolism signaling, LPS/IL-1-mediated inhibition of RXR function, and LXR/RXR activation) were inhibited by *Salmonella *infection. Two pathways most related to cell growth and proliferation (IL-9 signaling and EGF signaling) were activated by *Salmonella *infection (Additional file 10 Figure S1 EGF and Figure S2 IL-9), but two pathways, Insulin receptor signaling and Estrogen receptor signaling related to cell growth and proliferation were inhibited. Three pathways most related to cell death and apoptosis (NRF2-mediated Oxidative Stress Response, p38 MAPK signaling and LPS-stimulated MAPK signaling) were activated.

**Figure 5:**
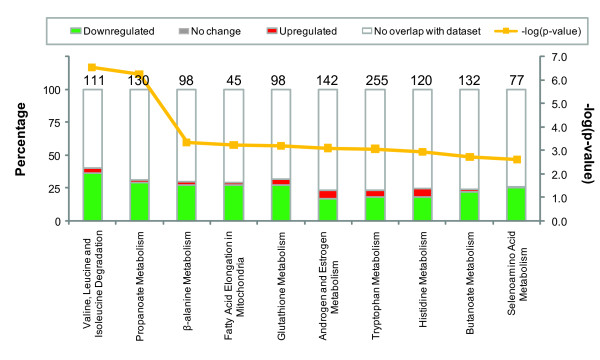
**The most 10 significant metabolism pathways associated with differentially expressed genes at 4 days post-infection**. The 10 most significant metabolism pathways across the entire dataset, and across multiple datasets, y-axis displays the significance. For the ratio, taller bars have more genes associated with the metabolism pathway than shorter bars metabolism pathway displays the number of up-regulated (red), down-regulated (green), and unchanged molecules (gray) in each pathway.

We identified canonical pathways involving metabolism that are associated with differential genes at 4 days post infection. Figure 5 Additional file 11 Table S11, and Additional file 12 S12 show most of the genes involved in these metabolic pathways and all the metabolism pathways involved in mouse mucosa infection. Valine, leucine, and lsoleucine degradation and carbohydrate metabolism are the two most significant pathways in the analysis list. The top function of valine, leucine, and isoleucine degradation involves lipid metabolism, molecular transport, and nucleic acid metabolism.

**Figure 6:**
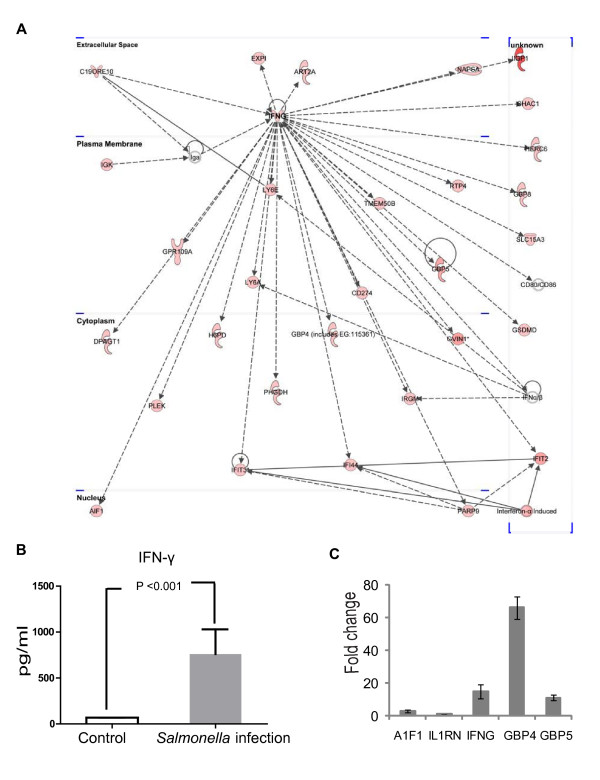
**Ingenuity pathway analysis of the IFN-γ network**. A. Ingenuity pathway Analysis network 2 depicting relationships among upregulated genes at 4 days post-infection. The intensity of the node color-(*red*)) indicated the degree of up-regulation. Edges are displayed with various labels that describe the nature of relationship between the nodes: ___ represents direct relationship; ----- represents indirect relationship; →represents acts on .Nodes are displayed using various shapes that represent the functional class of the gene product same as shown Figure 6. B. Serum IFN-γ levels in the control and the *Salmonella *infection group 4 days postinfection. C. Real-time PCR results for some targeted genes from the IFN-γ network.

**Figure 7:**
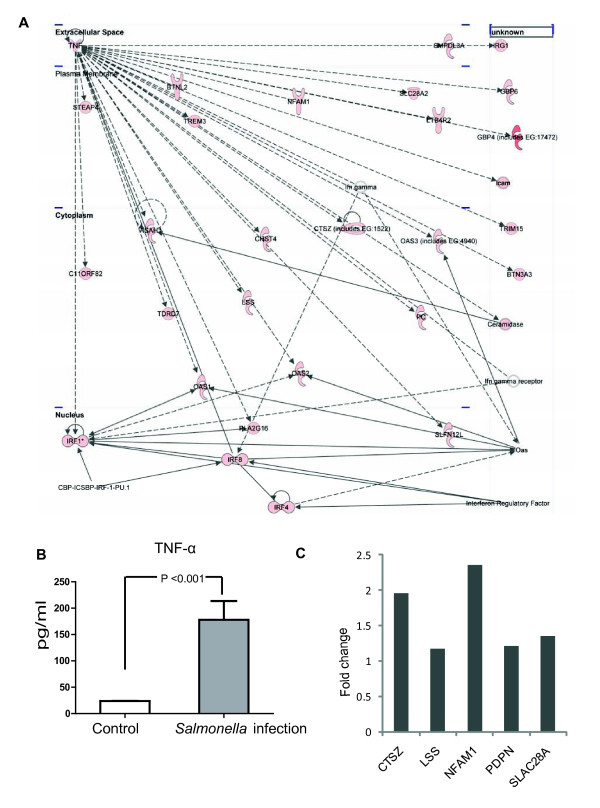
**Ingenuity pathway analysis of the TNF-α network**. A. Ingenuity pathway Analysis network 3 depicting relationships among upregulated genes at the 4 day post-infection. Intensity of the red color indicates the degree of up-regulation. Edges are displayed with various labels that describe the nature of relationship between the nodes: ___ represents direct relationship; ----- represents indirect relationship; →represents acts on. Nodes are displayed using various shapes that represent the functional class of the gene product same as shown Figure 6. B. Serum TNF-α level in the control and the *Salmonella *infection group at 4 days post infection. C. Real-time PCR results for some targeted genes from the TNF-α network.

Moreover, we identified 23 highly significant networks of interacting genes from amongst the up-regulated genes at 4 days post-infection group (Additional file 13 Table S13). For down-regulated genes, we identified 23 networks (Additional file 14 Table S14). The two highest ranked networks, IFN-γ and TNF-α, are provided in Figure 6 and Figure 7.

##### IFN-*γ*

Network 2 presents IFNG (IFN-γ) in central positions and consists of 35 DEGs genes that are all regulated positively by IFN-γ (Figure 6A and Additional file 15 Table S15). The network is correlated with following functions: cell growth and proliferation (*C190RF10, IFNG, LY6E, ATF1 *and *IRGM*), inflammatory response (*IFNG, IRGM, CD274 *and *IFI44*), lipid metabolism *(IFNG, DPAGT1, PHGDH*, *H6PD, GBP5*, and *GPR109A*), and small molecule biochemistry (*IFNG, DPAGT1, PHGDH, LY6E, H6PD, GBP5*, and *GPR109A*). Microarray data showed that all genes in the network were prominently up-regulated.

In order to confirm the physiological relevance of IFN-γ *in vivo*, we further investigated the secretion of the IFN-γ cytokine in mouse serum. In Figure 6B, significant differences were found between control and 4 days post-infection. Using real-time PCR, we further verified the expression of some genes in the IFN-γ network (Figure 6C). *IFNG, GBP4*, and *GBP5 *showed dramatic increase post *Salmonella *infection.

##### TNF-α

The network3 presents TNF (TNF-α) in central positions and consists of 28 DEGs genes that are all positively regulated by TNF-α (Figure 7A and Additional file 16 Table S16). The network is linked to the following functions: nucleic acid metabolism (SLC28A2), lipid metabolism (*PDPN, ASAH1, LSS*, *NAMPT *and *TNF*), small molecule biochemistry (*ASAH1, LSS, NAMPT, TNF and SLC28A2*), cell death (*NAMPT, TNF, CHI3L1 *and *ASAH1*), hematological system development and function (*CHST4, TNF and SLFN12L*), immune cell trafficking (*CHI3L1, TNF *and *CHST4*), cellular function and maintenance (*CTSZ *and *TNF*), genetic disorder (*ASAH1 *and *BTNL2*), inflammatory disease (*BTNL2 *and *TNF*), antigen presentation (*CTSZ *and *TNF*), cellular growth and proliferation (*C11ORF82, SLFN12L *and *TNF*), and inflammatory response (OAS1 and TNF).

We also investigated the secretion of the TNF-α cytokine in mouse serum. In Figure 7B, significant difference was found between control and 4 days post infection. Moreover, the expressions of some genes from the TNF-α network were verified using real-time PCR assay (Figure 7C).

##### NR3C1

**Figure 8:**
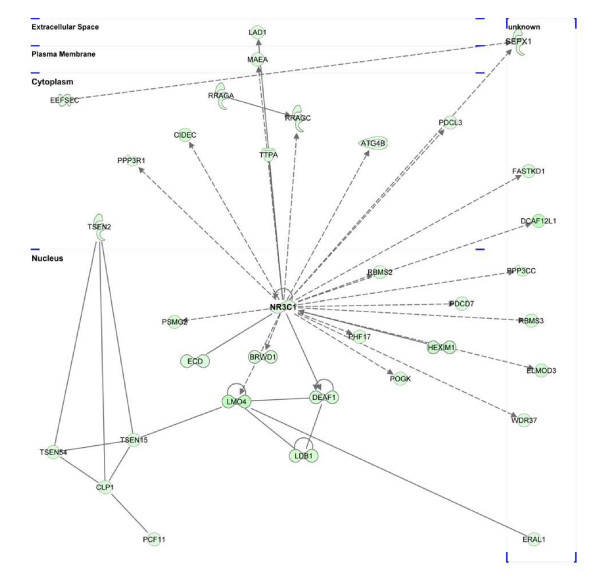
**Ingenuity pathway analysis network 4 depicting relationships among down-regulated genes at 4 days post-infection**. Intensity of the green color indicates the degree of down-regulation. Edges are displayed with various labels that describe the nature of relationship between the nodes: ___ represents direct relationship; ----- represents indirect relationship; →represents acts on. Nodes are displayed using various shapes that represent the functional class of the gene product same as shown Figure 4.

The NR3C1 protein is a receptor for glucocorticoids that can act as both a transcriptional factor and as a regulator of other transcription factors. As shown in Figure 8 and Additional file 17 Table S17, NR3C1 was in the central position of the network. This network was consisted of 35 down-regulated genes that are all positively regulated by NR3C1. We observed that top functions these genes involved are RNA post-Transcriptional Modification (*CLP1 TSEN2 *and *TSEN54*) and infectious disease and inflammatory disease (*NR3C1, PPP3CC*, and *PPP3R1*). Down-regulation of this gene is caused by glucocorticoid resistance, or cortisol resistance. Currently, glucocorticoid resistance or insensitivity is a major barrier in the treatment of several common inflammatory diseases [68].

### Co-regulated biological and common pathways at both times post *Salmonella *infection

The IPA analysis confirmed the major functionally related groups that were found to be commonly up-or down-regulated in both of the time-point infection groups. Thus, the pathways functionally linked to cell function and maintenance were induced at both the early stage and late stage of infection (Figure 2A and 2C). The pathways functionally related to cell cycle control and gene expression displayed suppression in *Salmonella *infection at both time points (Figure 2B and 2D), whereas the pathway functionally related to lipid metabolism showed up-regulation in response to *Salmonella *infection at early stage, but showed down-regulation at the late stage of 4 days post infection.

To investigate the co-regulated biological processes during the early and late stages of SL1344 infection, we searched for co-differentially expressed genes among both groups. A set of 43 genes was found to be commonly up-regulated in response to *Salmonella *infection at the early and late stage of infection (Additional file 18 Table S18). The top five functional categories associated with these common up-regulated genes are as follows: cellular assembly and organization (*DYNLT1*, *ANXA1*, *BARD1*, *SRF*, *ANXA2*, *ENG *and *TMSB10*); cellular development (*MUC1*, *DYNLT1*, *PCSK9*, *ANXA1*, *SRF*, *CAPG*, *JUNB*, *ENG *and *PLAC8*); gene expression (*MUC1*, *IL18RAP*, *SRF*, *JUNB*, and *ENG*); cell growth and proliferation (*MUC1*, *IL18RAP*, *BARD1*, *RRM2*, *SRF*, *ANXA2*, *JUNB*, *FGFBP1*, *ANXA1*, *CYBB*, *ENG*, *PLAC8 *and *TMSB10*); cell death (*MUC1*, *BARD1*, *CYBB*, *GMFG *and *TMSB10*) (Additional file 19 Table S19). Interestingly, Mucin 1 (*MUC1*) was found to be involved in a variety of biological processes, and *MUC1*'s role in host defense has been revealed by McAuley JL et al (2007)[69].

A set of 173 genes was found to be commonly down-regulated in response to *Salmonella *infection at both the early and late stage (Additional file 20 Table S20). The top five functional categories associated with these common down-regulated genes are shown in Additional file 21 Table S21. They are as follows: cellular development (*KITLG*, *CBX7*, *SLC4A2*, *TBX3*, *STRBP*, SAFB, *ROBO2*); cellular compromise (*KITLG*, *PRSS23, NFE2L2*); cellular movement (*KITLG*, *NOV*, *NDEL1*, *LPAR1*, *HSF2*, *ROBO2*, *NFE2L2*, *SLC12A6*); cell cycle (*KITLG, BHLHE40, HSF2, CHKA, SSTR1, MNAT1*); RNA post-transcriptional modification (*YTHDC1*, *RBM16*, *PNN*, *RPS28*, *SAFB*, *RPL26*, *EXOSC7*). Remarkably, *KITLG *was also found to be involved in a variety of biological processes (Table S7). The role of *KITLG *in the maintenance and survival of hematopoietic stem cells and of mast cells is well recognized [70] and was believed to play a role in tumorigenesis [71]. Downregulation of *KITLG *may inhibit cell migration and stem cell hemtopoiesis during the whole infection process.

In order to identity co-regulated pathway, we further performed pathway analysis. For these differentially expression genes between 8 hours pos-infection and 4 days post-infection, the metabolic pathways are shown in Figure S3 and the top canonical signaling pathways are in Figure S4. All of canonical signaling and metabolic pathway are listed in Tables S11 and S18. Comparing to 8 hours post-infection group, oxidative phosphorylation, ubiquinone biosynthesis, and mitochondrial dysfunction were totally shut-off at the 4 days post infection (Additional file 10 Figure S3 and S4). As shown in Table S18 (Additional file 18), most of signaling pathway list is similar to that of the pathway analysis for these genes between the 4 days post-infection group and the control group. The early modified signaling pathways, such as p53 signaling, was not maintained 4 days post infection. Moreover, pathway comparison analysis for the data of the 8 hours post-infection relative to control with the 4 days post-infection relative to control confirmed these results (Additional file 10 Figure S5).

### Validation of differentially expressed genes by real-time-PCR

**Figure 9:**
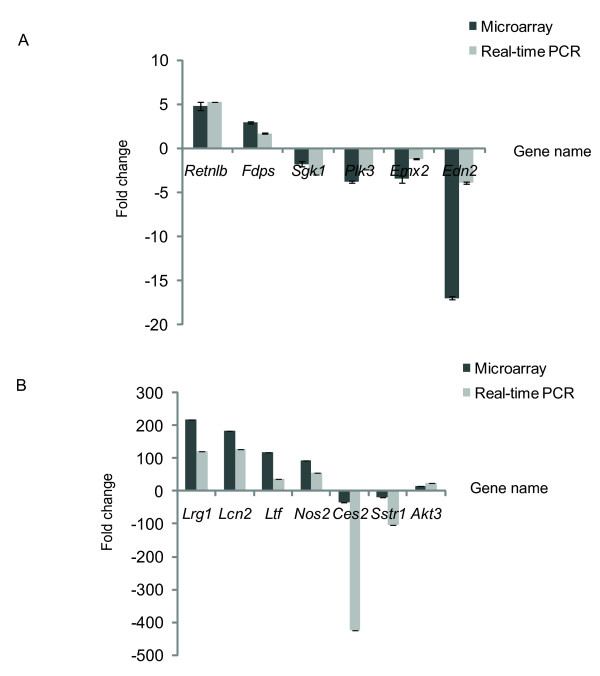
**Real-time PCR analysis and microarray comparison**. Transcript levels for genes of interest were determined in triplicates with control group and infection group at 8 hr post-infection or 4 days post-infection and normalized to β-*actin *transcript levels. Expression level of these interest genes derived from control group were set to one. Accordingly, relative fold times at 8 hours post-infection or 4 days post-infection were normalized to control group. At each time point, error bars indicate. the standard deviation: mean ± SEM (n = 3). A. real-time PCR analysis and microarray comparison at the early stage of Infection. B. real-time PCR analysis and microarray comparison at the late stage of infection.

To validate the microarray results, we analyzed 13 transcripts, in addition to the 10 genes from the IFN-γ and TNF-α networks, by quantitative real time-PCR (Figure 9). These genes were selected because of their top ranking positions on the differentially expressed gene list at both time points. Results showed that all of genes exhibited a similar transcriptional profile to that of microarray data. The Pearson correction coefficient between the qRT-PCR and microarray data for 13 top ranking differentially expressed was 0.86. Moreover, 10 genes with lower or medium fold change around both INF-γ and TNF-α network (Figure 6 and Figure 7) were also analyzed using samples generated from infected animals. Real-time PCR results showed 9 genes were up-regulated with the similar transcriptional profile as that of microarray data, except IL1RN without any change. Hence, the microarray provided a reliable comparison of gene expression in mouse mucosa samples at 8 hours and 4 days post-infection.

### Validation of differentially expressed genes at the protein level by Western blot

Akts are crucial mediators of various cellular processes, such as cell proliferation, apoptosis, regulation of the cell cycle and metabolism, and protein synthesis. Pathway analysis indicated that Akt3 is involved in the following pathways, including NF-κB pathway, EIF2 signaling, Glucocorticoid receptor signaling, eIF4 and p70S6K signaling, IL-4 signaling, Insulin receptor signaling, mTOR signaling, Jak/Stat Signaling, and VEGF signaling (Additional file 5 Table S5). In order to confirm Akt's function in *Salmonella *infection, we further analyzed Akts protein expression level using Western blot and immunofluorescence.

**Figure 10:**
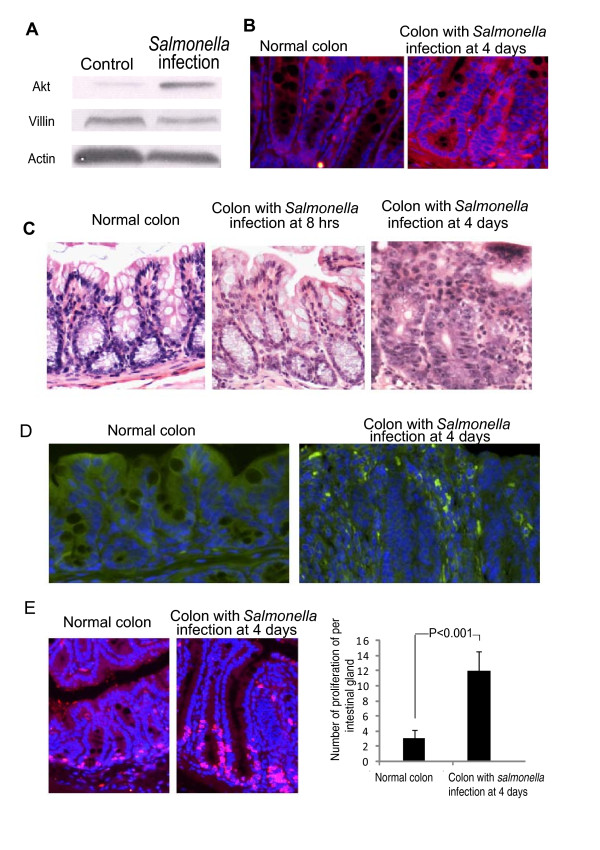
**Biological effects in colon mucosa with *Salmonella *infection**. A. Expression of Akt protein with or without *Salmonella *infection at 4 days post-infection. B. Distribution of Akt in normal or infected mouse colon at 4 days post-infection. C. Representative pathological aspects of colon section in the control, the 8 hour post-infection group, and the 4 day post infection group. Please note that *Salmonella *induce inflammatory features at 4 days post-infection. D. *Salmonella *distribution in mouse colon 4 days post-infection *Salmonella *shown in bright green in the mouse mucosa by using immuno-staining assay. E. BrdU labeling of small intestine epithelial cells showed *Salmonella *increase colon intestinal epithelial cell proliferation *in vivo*. The number of BrdU-positive cells per three high powered fields was counted. *n *= 3 in each experimental group.

As analyzed by Western Blot (Figure 10A), *Salmonella *infection increased the expression of total Akt proteins compared to the control. This result is in agreement with changes at the mRNA expression level. An important step in Akt activation is its translocation from the cytosol to the plasma membrane [72]. Therefore, we tested whether Akt became activated in response to the infection of *salmonella *in colon mucosa. We found that the total Akt protein was located in cytosol of the normal colon. In contrast, most of the Akt was translocated in the plasma membrane with stronger staining in the infection group (Figure 10B bright red staining).

### Histopathological analysis of *Salmonella*-infected and non infected tissues

To confirm the *Salmonella*-induced colon mucosal inflammation, we performed histopathological analysis of H&E-stained tissue sections. As shown in Figure 10C, we did not observe inflammatory pathological changes in the infection group at 8 hours compared to the control group. Both the infection group at 8 hours and control group showed the integrity of the epithelial layer identical to that of control group. However, at 4 days post infection, H&E-stained tissue sections revealed extensive pathological changes in the colon epithelium. We observed several inflammatory features, including crypt destruction and villin degradation, as well as the presence of necrotic epithelial cells (Figure 10C). In addition, immunostaining data also showed the presence of *Salmonella *in mouse colon 4 days post-infection (Figure 10D).

Gut homeostasis is maintained through a balance between cell damage due to the collateral effects of bacterial killing and epithelial repair by proliferation. At the late stage of infection, a series of pathways associated with inflammatory response and proliferation were identified (Table 2). Next, we examined the biological effect of *Salmonella *infection expression on epithelial proliferation, which is regulated by multiple pathways, including the Akt and EGF pathways. BrdU staining was performed to measure the BrdU incorporation into newly synthesized DNA. As shown in Figure 10E, BrdU-positive staining (pink) in the merged BrdU staining (red) and DAPI (blue) showed that *Salmonella *infection induced a more dramatic increase in epithelial cell proliferation compared to the control group without any treatment. The number of the proliferating cells per intestinal gland further showed that *Salmonella *increased epithelia proliferation to 12 proliferative cells per intestinal gland (Figure 10E). Our biophysiologic data is consistent with the microarray pathway analysis.

## Discussion

In the current study, the *Salmonella*-induced pathway and network changes were mainly observed to show inflammatory inhibition and oxidative stress in mitochondria at the early stage of infection, while at the late stage of infection, the dramatic changes in thousands of gene expression are characterized.

Two networks for up-regulated genes around IFN-γ and TNF-α were identified and cross-talked with some identified signaling pathways. Furthermore, a series of pathways associated with inflammatory/immune response, cell proliferation, cell apoptosis, and developmental disorder were appraised. The biochemical and pathologic data were consistent with the microarray analysis and confirmed the biological role of *Salmonella *in inducing inflammation and epithelium cell proliferation through the regulation of multiple signaling pathways.

### *Salmonella *infection and apoptosis

Intestinal epithelial apoptosis is a response to bacterial infection [73]. *Salmonella *effector AvrA dampened the proapoptotic innate immune response to *Salmonella *at the mouse intestinal mucosa [74]. Our microarray data also showed that a number of genes involved in apoptosis presented *Salmonella*-induced expression changes, including up-regulated Caspase family members (*CASP3, CASP7*, *CASP8 and CASP12*), Poly (ADP-ribose) polymerase family members *(PARP3, PARP9, PARP12 and PARP14*) and some down-regulated genes (*Famim3, Stk4, Stk17 and Nalp1*). Accordingly, as shown in Table 2 strong induction of apoptosis-related pathways were involved in response to *Salmonella *infection at 4 days, such as IL-9 (anti-apoptosis), retinoic acid mediated apoptosis (pro-apoptosis), caspase family mediated apoptosis(pro-apoptosis), and LPS-stimulated MAPK pathway (pro-apoptosis). These apparently contradictory pathways may reflect the complexity of the apoptosis process in mouse colon mucosa responded to *Salmonella *infection.

*Salmonella *effector protein SigD/SopB protects epithelial cells from apoptosis by sustained activation of Akt [75,76]. Our microarray analysis along with the Western blots and immunostaining *in vivo *confirmed these previous researches. Overall, these results suggest that *Salmonella *infection *in vivo *increased Akt protein levels and induced Akt activation, thus regulating multiple signaling pathways.

### Epidermal growth factor receptor (EGFR) is involved in *Salmonella *infection *in vivo*

EGFR is a transmembrane glycoprotein with an intrinsic tyrosine kinase. Ligand binding to the EGFR activates cell signaling. Galan *et al*. (1992) [77] reported that stimulation of the EGF receptor is involved in the invasion of cultured Henle-407 cells by *Salmonella *infection. EGFR downstream signaling proteins initiate several signal transduction cascades, principally the Stat3/Stat1, MAPK, Akt and JNK pathways, leading to DNA synthesis and cell proliferation [78]. Bertelson *et a*l. further reported *Salmonella *effector SigD can activate the EGFR signaling in T84 epithelial monolayers cells through downstream signaling PI3K, and pointed out that this activation may induce different actions than what is observed in the EGFR pathway [79]. We observed the mRNA level of *EGFR *to be up-regulated (Additional file 22 Table S22 and Figure S1), and downstream signaling protein, such as STAT3, STAT1, AKT3 and MKK4 also showed up-regulation at 4 days post infection. Hence, our microarray data confirms previous research and extends the down-stream signaling of EGFR response to *Salmonella *infection and provides more comprehensive information about the EGFR pathway involved in *Salmonella *infection.

### Oxidative stress response signaling and metabolism

NRF2-mediated oxidative stress response signaling was the most significant pathway at 4 days post infection (Table 2). This pathway involved 55 up-regulated genes and 24 down-expressed genes. Oxidative stress is caused by an imbalance between the production of reactive oxygen and the detoxification of reactive intermediates. Severe oxidative stress can trigger apoptosis and necrosis. The cellular defense response to oxidative stress includes induction of detoxifying enzymes, heat shock proteins, and antioxidant enzymes (microarray data suggested). Roland Nilsson et al found that LPS stimulation is a pivotal role for NRF-2 in orchestrating the LPS response in macrophages [80]. NRF2-mediated oxidative stress response signaling in the mouse colon intestine may be activated by *Salmonella *LPS. Interestingly, heat shock protein 40 (DNJ family) showed significant change in this pathway (Additional file 23 Table S23). Of the DNJ members, DNJ5 showed the most significant up-regulation. Takaya A et al (2004)[81] reported that DnaK/DnaJ chaperone machinery is involved in the bacterial invasion of intestine epithelial cells. Recently, ERdj3, an endoplasmic reticulum luminal chaperone of the Hsp40/DnaJ family, is further indentified as a target for *Salmonella *effector protein SlrP in HeLa cells [82]. Taken together, *Salmonella *effector Slrp may play a role in transmitting NRF2-mediated oxidative stress response signaling in colon mucosa.

As shown in Additional file 10, Figure S6 and Additional file 24 Table S24, all genes involved in antigen presentation pathway were up-regulated. These results are consistent with the gene expression patterns observed in the porcine lung during *Salmonella *infection [12]. These data illustrate that the antigen processing pathway was activated by pathogenic *Salmonella *infection in colon mucosa (Table 2 Figure S6).

Most genes, such as *CD80*, *FAS, PLA2G12A, PLA2G12B, PTGS, TNFRSF1A, TNFSF1B, IIGP1*, *TRAF1, TIRAP, AKT3, TLR2, TLR3, TLR4, STAT1, STAT2, SLC11A1, SLC16A3, IRF9 IFIT2, and IL1B*, which are known to be involved in innate/inflammatory pathway, increase their RNA expression levels significantly at 4 days post-infection. Accordingly, p38 MAPK signaling, MIF regulation of innate immunity, and LPS-stimulated MAPK signaling pathways were all activated. Most of interferon-induced protein, such as *IFI35*, *IFI73*, *IFNAR2 *and *IFNG*, were up-regulated by *Salmonella*. At 4 days post-infection, interferon signaling pathways were strongly affected (Table 2 Additional file 10 Figure S7 and Additional file 15 Table S15). Top functions of these genes were associated with antigen presentation, cell morphology and cell to cell signaling.

As shown Additional file 25 Table S25, 43 enzymes in the valine, leucine, and isoleucine degradation pathway were down-regulated at 4 days post-infection, including acetyl-Coenzyme A acyltransferase family member (*ACAA1, ACAA2 *and *ACAA1B*), acyl-Coenzyme A dehydrogenase family member (*ACAD8, ACAD10, ACADL, ACADM *and *ACADS*), and aldehyde dehydrogenase family member (*ALDH2, ALDH1A1, ALDH1A3, ALDH1A7, ALDH1B1, ALDH1B1, ALDH3A2 *and *ALDH6A1, ALDH7A1*). Interestingly, we observed that these enzymes are also involved in other metabolic pathways including valine, leucine, and isoleucine degradation, propanoate metabolism, fatty acid metabolism, and fatty acid elongation in mitochondria. Thus, down-regulation of these important genes may play key role in disordering embolism activities of colon mucosa.

### NF-κB

NF-κB is a key transcriptional regulator of innate and adaptive immunity. We found that *S100A1*, *MUC1*, *and TRIP6 *around NF-κB increases NF-κB activity, but rather, *BEX2, GLRX3, GPX1 *and *PXCARD *decreases NF-κB activity. Interestingly, our microarray data showed that the expression level of *BEX2, GLRX3, GPX1 *and *PXCARD *were down-regulated at 4 days post-infection, which is different from the up-regulated mRNA level at 8 hours post infection. However, *S100A1, MUC1*, and *TRIP6 *showed a continued up-regulated status at 4 days post-infection.

*I*κ*Bα *and *I*κ*Bz *as inhibitory genes are activated by NF-κB in a negative feed back loop, which provides an effective mechanism for controlling the NF-κB activity [83,84]. However, we found both genes were not indentified in this network. Further microarray data also showed mRNA level of *I*κ*Bα *and IκBz remained unchanged at 8 hours post infection, but showed prominent change at 4 days post infection.

Based on the above microarray information, we speculate that NF-κB activity undergoes early stimulation without demonstrable feedback regulation, but at with demonstrable feedback regulation at the late stage of infection. Porcine MLN during *Salmonella *infection also showed the similar regulation process [12].

### IFN-γ and TNF-α

IFN-γ is a remarkable cytokine that orchestrates many distinct cellular programs through transcriptional controlling over large numbers of genes [85]. The role of IFN-γ is related to host defense against *Salmonella *infection [86]. Actually, the network analysis supports that interferon signaling was activated by *Salmonella *infection. We further pointed out the central role that IFN-γ plays in mice colonic against bacterial infection (Figure S7 and Table S15).

GTPase family is clearly regulated by IFN-γ-induced genes [87], which regulate the survival of pathogens residing in phagosomes vacuoles. We observed that GTPase family members, such as *GViN1, Gbp8, Gbp5, IIGP1 *and *IRGM*, are directly targeted by IFN-γ (Figure 6A). The data correlate with the observation in rat colonic cells infected with *Salmonella *[58,88]. In particular, *IIGP1 *was found to be highly up-regulated in our microarray data (90 fold times). Uthaiah RC et al (2003) [89] also reported that recombinant IIGP1 showed cooperative enzymatic activity and GTP-dependent multimerization.

TNF-α encodes a multifunctional proinflammatory cytokine that belongs to the tumor necrosis factor (TNF) superfamily. This cytokine is involved in the regulation of a wide spectrum of biological processes including cell proliferation, differentiation, apoptosis and lipid metabolism [90,91]. As expected, the genes in this network are associated with TNF function. Interestingly, we observed *GBP4 *and *GBP6 *as IFN-γ induced genes (Figure 6A) that are also involved in TNF-α network. *GBP4 *showed highly up-regulated in microarray data. Degrandi *et al*. (2007)[92] reported mouse TNF-α protein increases expression of mouse *GBP4 *mRNA in ANA-1 cells, but we did not find other reports showing that *GBP6 *were TNF-α-induced genes. Therefore, further experiment is needed to establish whether this gene is up-regulated by TNF-α in mouse colonic mucosa after *Salmonella *infection.

Clare *et al*. (2003) used ICAM (-/-) knockout mice to demonstrate that ICAM-1 plays a critical role during the rechallenge of immunized mice with virulent *Salmonella *[93]. Our network and microarray data also confirmed that the intracellular adhesion molecule ICAM was induced by TNF-α. We further observed *CTSZ *as an antigen presentation molecule is also up-regulated. Thus, the network analysis is consistent with the previous experiment results: production of TNF-α in the intestinal tract following *S*. *typhimurium *infection and the observation that early pathology induced by *Salmonella *infection of the gastrointestinal tract is mediated by immune mechanisms [94].

Overall, the number of connections among the molecules other than TNF-α or IFN-γ is quite limited (Figure 6A and Figure 7A). Most of genes are targeted directly by TNF-α or IFN-γ, which are very different from that of NF-κB network shown in Figure 4. Hence, these TNF-α or IFN-γ networks further reflect the pleiotropic action of proinflammatory cytokine involved in host defense against *Salmonella *infection by regulating extensive biological process.

### T helper 2 (Th2) immune response

Both Interleukin-4 (IL-4) and Interleukin-9 (IL-9) are multifunctional cytokine secreted by T helper 2 (Th2) lymphocytes. IL-9 stimulates the growth and proliferation of T cells, and promotes the proliferation and differentiation of mast cells and hematopoietic progenitors [95,96]. IL-4 plays a critical role in the regulation of immune responses [97] and the pathogenesis of inflammatory bowel disease [98,99].

Previous research study reveled that IL-9 receptor and IL-4 receptor ligation results in auto and/or trans-phosphorylation of Janus kinases 1 and 3 (JAK1 and JAK3) phosphorylation of the receptor, and activation of the pathways involved in IL-9 signaling and IL-4 signaling [100,101]. These pathways include signal transducer and activator of transcription 1, 3, 5 and 6 (STAT1, STAT3, and STAT5 and STAT6), Insulin receptor substrate 1 and 2 (IRS-1 and IRS-2)/Phosphoinositide-3-kinase (PI3K regulatory subunit) and Extracellular signal regulated kinases 1 and 2 (ERK1/2) [102-104].

We observed the mRNA level of IL-9 receptor (*IL-2 R*) and IL-4 receptor (*IL-2R, IL-4R IL-13 R*) are up-regulated and that downstream signaling protein, such as JAK2 JAK3, STAT1, STAT2, STAT3, IRS1, SOCS1 and SOCS3 showed up-regulation at 4 days post infection (Figure S2, Figure S8, Additional file 26 Table S26, and Additional file 27 Table S27). Dumoutier *et al*. reported that STAT1 and STAT3, activated by IL-9, then up-regulate the transcription of IL-3 and IL-22, which are involve in the generation of inflammatory and allergic responses [95]. Accordingly, we also observed that Interleukin-3 and 22 were up-regulated in mouse colon mucosa with *Salmonella *infection at four days (Additional file 2 Table S2). IL-4 is produced in response to IL-18 or IL-33 stimulation from mouse basophils [105]. We also found IL-18b and IL-33 to be up-regulated (Additional file 2 Table S2). Overall, these data illustrate that the IL-4 and IL-9 signaling pathway associated with TH2 immune response was activated by pathogenic *Salmonella *infection in colon mucosa.

Recent advances have called attention to the the involvement of allergen- and parasite product-mediated activation of epithelial cells, basophils and dendritic cells and the functions of the cytokines IL-4, IL-25, IL-33 in the initiation and amplification of TH2-type immune responses *in vivo *[106,107].

Cytokines play a key role in IBD that determine T cell differentiation of Th1, Th2, T regulatory and newly described Th17 cells [99]. Hence, IL-4 and IL-9 signaling pathway activated in mouse mucosa with *Salmonella *infection provides more comprehensive information about how the Th2 immune system interplays with signaling transducers in colon mucosal inflammation.

In *Drosophila*, the Janus kinases-signal transducers and activators of transcription (Jak-Stat) pathway plays an important role in hematopoiesis, stress response, stem cell proliferation, and antiviral immunity in intestine [87,108-110]. Interestingly, mouse microarray data showed Jak2, Stat1 and Stat3 as vital proteins in this pathway and were up-regulated at the 4 days post-infection. The mouse colon mucosal complex system is different from *Drosophila *gut, stat proteins are intracellular effector molecules of cytokine-modulated signaling in mammalian immune system [111]. Further research is needed to validate our analysis and how JAK-Stat signaling regulates the host response during *Salmonella *infection.

However, even if we confirmed the coherence of our microarray data by other molecular biology approaches, this study has limitations: transcriptional changes not representing the changes at the post-transcriptional level, posttransductional behavior of the differentially expressed genes, and statistical error. For example, our published data showed that *Salmonella *effector AvrA can activate the beta-catenin pathway through deubquitination [44]. However, this activated pathway was not revealed in this analysis. Further studies combining genomic and proteomic are necessary to find out more details of host cell interplay with *Salmonella*. Moreover, the colon mucosa tissue samples used in this experiment contained several cell types. Diverse pathways may be activated in different cell types, not necessarily within one kind of cells. Future research on structured information and pathways occurring in individual kind of cells is required.

## Conclusion

In this current study, we are the first to show a bioinformatics strategy to investigate global pathway and network of host responses to *Salmonella *infection in mouse colon mucosa at the early and chronic infection stages. We found that *Salmonella *infection caused dramatic changes in gene expression of colon mucosa, which further leads to a sequence of cellular events that involve activating and blocking signaling modulation responses in colon mucosa. IFN-γ- and TNF-α receptor-mediated signaling cascades stimulated the expression of IFN-γ- and TNF-α-inducible genes. We not only confirmed IFN-γ and TNF-α secretion in mice infected with *Salmonella*, but also observed that many of the genes regulated by cytokine IFN-γ and TNF-α contributed to the modulation of cell proliferation and growth, apoptosis, and developmental disorders.

Moreover, we observed a general repression process of metabolic pathways, specifically shown in the amino acid metabolic and lipid metabolic related pathways. These changed genes are not commonly altered in a cytokine-dependent manner. We speculate that the inhibited metabolic pathways in host cells combined with the relevant signaling pathways presumably increase the opportunities of bacteria growth in host cells, and can further lead to metabolic, infectious, and inflammatory diseases in the intestine.

Overall, our data provide not only new networks between the genes for understanding the biologic properties of *Salmonella *infection in mouse colon mucosa, but also provide useful pathway maps for future understanding of the pathology of inflammatory bowel diseases, inflammation associated colon tumorigensis and other diseases. It will help us to develop a new protocol for anti-bacterial infection, risk assessment, and prevention of the intestinal illness and other chronic diseases.

## Abbreviations

DEG: Differentially expression gene; EGF: Epidermal growth factor; EGFR: Epidermal growth factor receptor; ERK: extracellular signal-regulated kinases; IBD: inflammatory bowel diseases; ICAM: intercellular adhesion molecule; IFNG: Interferon-gamma; IPA: Ingenuity Pathways Analysis; IL-4: Interleukin-4; IL-9: Interleukin-9; JAK-STAT: Janus kinases- Signal Transducers and Activators of Transcription protein; JNK: JUN-NH_2_-terminal kinase; LPS: Lipopolysaccharides; MAPK: mitogen-activated protein kinase; MTOR: mammalian target of rapamycin; NF-κB: Nuclear factor κB; PXR: pregnane × receptor, or NR1I2 (nuclear receptor subfamily 1, group I, member 2); RXR: Retinoid × receptor; TNF: Tumor necrosis factor; TTSS: Type Three Secretion System; STAT: Signal transducer and activator of transcription.

## Authors' contributions

XL: participated in experimental design, animal experiment, preparation of RNA sample, real-time PCR, western blot and immunofluorescence analysis, acquisition of data, analysis and interpretation of data, carried out bioinformatics analysis, and drafted table, figure and the manuscript.

RL: participated in experimental design, analysis and interpretation of data, real-time PCR analysis, drafted tables and figures, and carried out animal experiments.

YX: participated in interpretation of data, performed statistical analysis, and edited the manuscript for important intellectual content.

JS: participated in study concept and design, acquisition of data, analysis and interpretation of data, material support, writing and critical revision of the manuscript for critical intellectual content, obtained funding, and supervised study.

All authors read and approved the final manuscript.

## Acknowledgements and Funding

We thank Dr. Constance D. Baldwin and Julia Militar at the University of Rochester for critical revising and editing of this manuscript, Xi Emma Li for her excellent technical support, and Jody Bown and Stephen L. Willie for helpful suggestion on microarray software. This work was supported by the National Institutes of Health (DK075386-0251, R03DK089010-01), the American Cancer Society (RSG-09-075-01-MBC), and the IDEAL award from the New York State's Empire State Stem Cell Board (N09G-279) to Jun Sun.

## Supplementary Material

Additional file 1 **Primer sequence for qRT-PCR**. Listing all primer sequences used in qRT-PCR (PDF file). PCR data were shown in Figure 6C, 7C, and Figure 9. Click here for file

Additional file 2 **The list of differentially expressed genes between the control and 8 hours post-infection groups**. Listing differentially expressed genes (fold times ≥ 1.2; P < 0.05) at 8 hours post-infection comparing with the control group without *Salmonella *infection. Click here for file

Additional file 3 **The list of differentially expressed genes between the control and at 4 days post- infection groups**. At 4 days post-infection, differentially expressed genes are listed in Table S3. Click here for file

Additional file 4 **The list of differentially expressed genes between the 8 hours and 4 days post- SL1344 infection**. Identify genes that were expressed differentially during two time course. Click here for file

Additional file 5 **All pathways involved in colon mucosa 8 hours post-SL1344 infection**. List pathways involved in colon mucosa with *Salmonella *infection at 8 hours. Click here for file

Additional file 6 **The gene list for Network 1, NF-kappaB**. List genes interact directly with NF-kappaB transcription factor. Click here for file

Additional file 7 **Networks of interacting up-regulated genes at 8 hours post-infection**. Identify 14 highly significant networks of potentially interacting up-regulated genes at 8 hours post-infection group. Click here for file

Additional file 8 **Down-regulated genes 8 hours post-infection**. Identify 16 highly significant networks of potentially interacting down-regulated genes and did not identify a network with center regulation. Click here for file

Additional file 9 **All signaling pathways involved in colon mucosa with *Salmonella *infection at 4 days**. List canonical pathways involving signaling associated with differential genes at 4 days post infection. Click here for file

Additional file 10 **Canonical pathways changed by SL1344 infection**. Figures for representative pathways involving signaling associated with *Salmonella *infection. Figure S1 EGF signaling; Figure S2 IL-9; Figure S3: Metabolic pathways; Figure S4: Top canonical signaling. Figure S5: co-regulated pathway between 8 hours and 4 days post-infection. Figure S6: Antigen presentation pathway; Figure S7: Interferon signaling; and Figure S8: IL-4 signaling. Click here for file

Additional file 11 **All metabolism pathways involved in colon mucosa with *Salmonella *infection at 4 days**. All metabolism pathways involved in colon mucosa with *Salmonella *infection at 4 days. Click here for file

Additional file 12 **All metabolic pathways involved in colon mucosa with these differentially expression gene between 8 hr post-infection and 4 day post-infection**. List the metabolism pathways involved in mucosa infection with these differentially expression gene between 8 hr post-infection and 4 day post-infection (fold times ≥ 1.2 times, p-value < 0.05). Click here for file

Additional file 13 **Networks of interacting genes from amongst the up-regulated genes at 4 days post-infection**. Identify highly significant networks of interacting genes from amongst the up-regulated genes at 4 days post-infection. Click here for file

Additional file 14 **Networks of interacting genes from amongst the down-regulated genes at 4 days post-infection**. Identify highly significant networks of interacting genes from amongst the down-regulated genes at 4 days post-infection. Click here for file

Additional file 15 **The gene list for Network 2**. Network 2 presents IFN-γ in central positions and consists of 35 DEGs genes that are all regulated positively by IFN-γ. Click here for file

Additional file 16 **The gene list for Network 3**. The network TNF-α in central positions and consists of genes that are all positively regulated by TNF-α. Click here for file

Additional file 17 **The gene list for Network 4**. The network 4 in central positions and consists genes that are all positively regulated by NR3C1. Click here for file

Additional file 18 **Common up-regulated genes function in both early stage and late stage of infection**. Identity commonly up-regulated in response to *Salmonella *infection at the early and late stage of infection. Click here for file

Additional file 19 **Functional categories associated with common up-regulated genes**. Identify the top functional categories associated with these common up-regulated genes. Click here for file

Additional file 20 **Common down-regulated genes function in both early stage and late stage of infection**. Identity commonly down-regulated in response to *Salmonella *infection at the early and late stage of infection. Click here for file

Additional file 21 **Functional categories associated with common down-regulated genes**. Identify the top functional categories associated with these common down-regulated genes. Click here for file

Additional file 22 **The gene list for EGFR signaling pathways at 4 days of post-infection**. EGFR and downstream signaling proteins are involved in *Salmonella *infection. Click here for file

Additional file 23 **The gene list for NRF2-mediated oxidative stress response signaling at 4 days of post-infection**. Click here for file

Additional file 24 **Antigen presentation pathway were up-regulated**. the antigen processing pathway was activated by pathogenic *Salmonella *infection in colon mucosa. Click here for file

Additional file 25 **43 enzymes down-regulated at 4 days post-infection**. 43 enzymes in the valine, leucine, and isoleucine degradation pathway were down-regulated at 4 days post-infection, including acetyl-Coenzyme A acyltransferase family member Click here for file

Additional file 26 **The gene list for IL-4 signaling pathway at 4 days of post-infection**. Identify genes associated with IL-4 at 4 days post-infection. Click here for file

Additional file 27 **The gene list for IL-9 signaling pathway at 4 days of post-infection**. Identify genes associated with IL-9 at 4 days post-infection. Click here for file
