# Supplementary material for: Global analysis of the eukaryotic pathways and networks regulated by Salmonella typhimurium in mouse intestinal infection in vivo
Source: BMC Genomics. 2010 Dec 20;11:722. doi: 10.1186/1471-2164-11-722 (PMC3022924; doi:10.1186/1471-2164-11-722)
Supplement: Additional file 1 — Primer sequence for qRT-PCR. Listing all primer sequences used in qRT-PCR (PDF file). PCR data were shown in Figure 6C, 7C, and Figure 9. [file 1471-2164-11-722-S1.PDF]

Supplementary Table1 (Table S1). Primer sequence for qRT-PCR

Gene name	Accession ID	Primer sequence	Product length(bp)
Lrg1	NM_029796	5'-CTGGATCTGACCCGCAATG-3'(F)	
		5'-CAAGGTCGAGGGTGTGCAG-3'(R)	226
Lcn2	NM_008491	5'-TGGCCCTGAGTGTCATGTG-3'(F)	
		5'-CTCTTGTAGCTCATAGATGGTGC-3'(R)	239
Ltf	NM_008522	5'-TGAGGCCCTTGGACTCTGT-3'(F)	
		5'-ACCCACTTTTCTCATCTCGTTC-3'(R)	112
Nos2	NM_010927	5'-ACATCGACCCGTCCACAGTAT-3'(F)	
		5'-CAGAGGGGTAGGCTTGTCTC-3'(R)	177
Akt3	NM_011785	5'-TGGGTTCAGAAGAGGGGAGAA-3'(F)	
		5'-AGGGGATAAGGTAAGTCCACATC-3'(R)	122
Ces1	NM_021456	5'-CTGCTTGCTTGAGTCTGGGAC-3'(F)	
		5'-TTGGTAGCACAAAGGAGGGTA-3'(R)	233
Sstr1	NM_009216	5'-CAGGGTAGCGCCATTCTCATC-3'(F)	
		5'-AGCGTGGAAGTGACCAGAAAG-3'(R)	194
Retnlb	NM_023881	5'-AAGCCTACACTGTGTTTCCTTTT-3'(F)	
		5'-GCTTCCTTGATCCTTTGATCCAC-3'(R)	110
Fdps	NM_134469	5'-GGAGGTCCTAGAGTACAATGCC-3'(F)	
		5'-AAGCCTGGAGCAGTTCTACAC-3'(R)	155
Sgk1	NM_011361	5'-CTGCTCGAAGCACCCTTACC-3'(F)	
		5'-TCCTGAGGATGGGACATTTTCA-3'(R)	175
Plk3	NM_013807	5'-GCACATCCATCGGTCATCCAG-3'(F)	
		5'-GCCACAGTCAAACCTTCTTCAA-3'(R)	154
Edn2	NM_007902	5'-CACCTGCGTTTTCGTCGATG-3'(F)	
		5'-CCAGTGTCTTCGATGGCAGAA-3'(R)	219
Emx2	NM_010132	5'-TCAGCTACGCCAATTCCAGTC-3'(F)	
		5'-ACCAAGTCCGGGTTGGAGTA-3'(R)	106
Beta-actin	NM_007393	5'-TGTTACCAACTGGGACGACA-3'(F)	
		5'-CTGGGTCATCTTTTCACGGT-3'(R)	139
LSS	NM_146006	5'-TCGTGGGGGACCCTATAAAAC-3'(F)	
		5'-CGTCCTCCGCTTGATAATAAGTC-3'(R)	104
NFAM1	AF361364	5'-CAGCCTTCCTATCATGGTGTCC-3'(F)	
		5'-GTCTTGTGAAGTCCTCGATTCTG-3'(R)	74
CTSZ	NM_022325	5'-GGCCAGACTTGCTACCATCC-3'(F)	
		5'-ACACCGTTCACATTTCTCCAG-3'(R)	134
PDPN	NM_010329	5'-ACCGTGCCAGTGTTGTTCTG-3'(F)	
		5'-AGCACCTGTGGTTGTTATTTTGT-3'(R)	159
SLC28A2	NM_172980	5'-AGTGGAGAATTGCATGGAGAAC-3'(F)	
		5'-GACCAAGCAGGATCTTTCTGAA-3'(R)	215
GBP4	NM_008620	5'-GGAGAAGCTAACGAAGGAACAA-3'(F)	
		5'-TTCCACAAGGGAATCACCATTTT-3'(R)	136
GBP5	AF487898	5'-TCTGTGGATCTCGCCTTTCC-3'(F)	
		5'-AGGTTGGACGGGTGACAGT-3'(R)	66
IL1RN	NM_031260	5'-GCTCATTGCTGGGTACTTACAA-3'(F)	
		5'-CCAGACTTGGCACAAGACAGG-3'(R)	155
AIF1	NM_019467	5'-ATCAACAAGCAATTCCTCGATGA-3'(F)	
		5'-CAGCATTCGCTTCAAGGACATA-3'(R)	144
TAP1	NM_019828	5'-GCTGGAGCTTTGCCTTATTGG-3'(F)	
		5'-GGTCAAAAAGGTCTCAGTGAACT-3'(R)	168
PSMB8	NM_010724	5'-ATGGCGTTACTGGATCTGTGC-3'(F)	
		5'-CGCGGAGAAACTGTAGTGTCC-3'(R)	111
PSMB9	NM_013585	5'-CATGAACCGAGATGGCTCTAGT-3'(F)	
		5'-TCATCGTAGAATTTTGGCAGCTC-3'(R)	111
