# Supplementary material for: Global analysis of the eukaryotic pathways and networks regulated by Salmonella typhimurium in mouse intestinal infection in vivo
Source: BMC Genomics. 2010 Dec 20;11:722. doi: 10.1186/1471-2164-11-722 (PMC3022924; doi:10.1186/1471-2164-11-722)
Supplement: Additional file 10 — Canonical pathways changed by SL1344 infection. Figures for representative pathways involving signaling associated with Salmonella infection. Figure S1 EGF signaling; Figure S2 IL-9; Figure S3: Metabolic pathways; Figure S4: Top canonical signaling. Figure S5: co-regulated pathway between 8 hours and 4 days post-infection. Figure S6: Antigen presentation pathway; Figure S7: Interferon signaling; and Figure S8: IL-4 signaling. [file 1471-2164-11-722-S10.PDF]

Figure S1

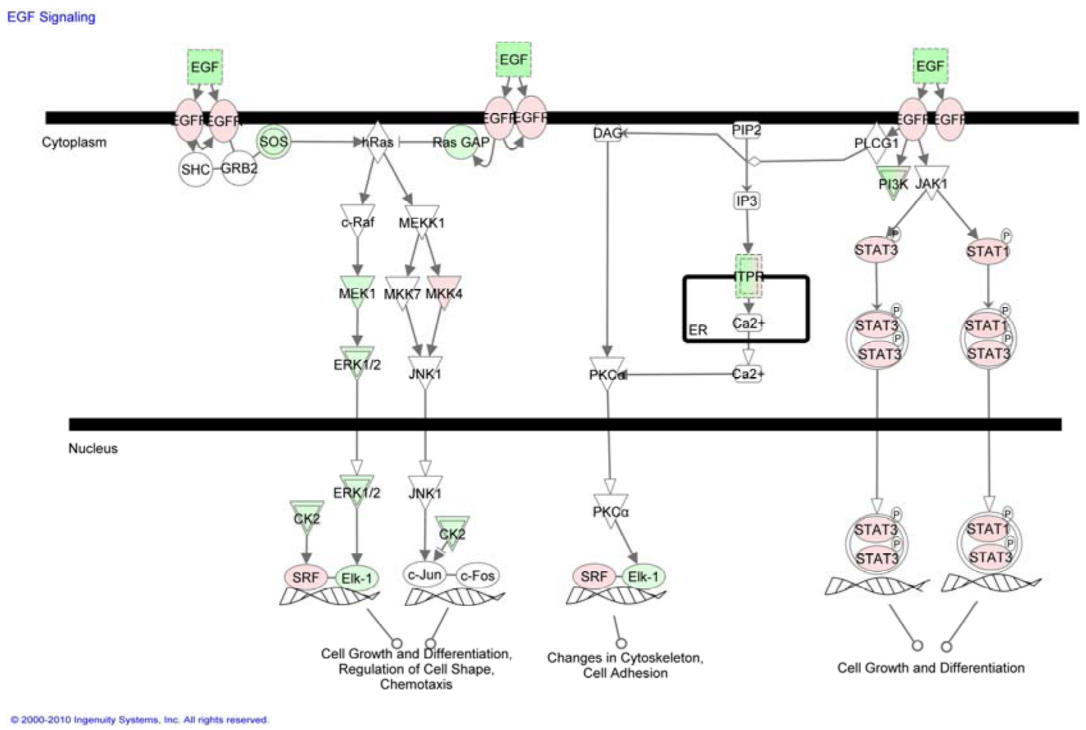
EGF Signaling
EGF
EGFR
Cytoplasm
SOS
SHC
GRB2
hRas
Ras GAP
DAG
PIP2
PLCG1
IP3
PI3K
JAK1
c-Raf
MEKK1
STAT3
STAT1
MEK1
MKK7
MKK4
ITPR
ER
Ca2+
ERK1/2
JNK1
PKCα
Nucleus
CK2
SRF
Elk-1
c-Jun
c-Fos
Cell Growth and Differentiation,
Regulation of Cell Shape,
Chemotaxis
Changes in Cytoskeleton,
Cell Adhesion
Cell Growth and Differentiation
© 2000-2010 Ingenuity Systems, Inc. All rights reserved.

Figure S2

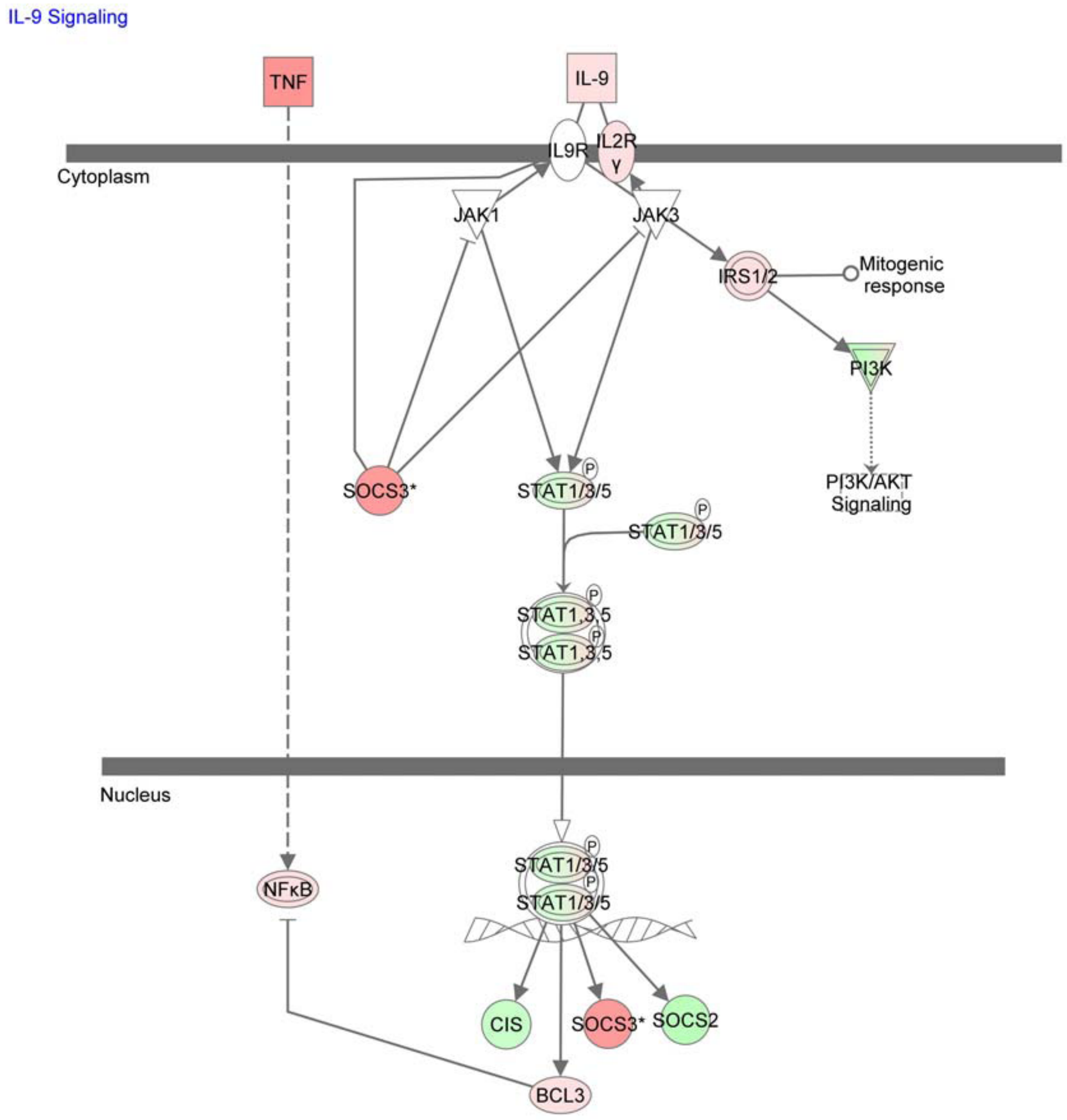

© 2000-2010 Ingenuity Systems, Inc. All rights reserved.

Figure S3

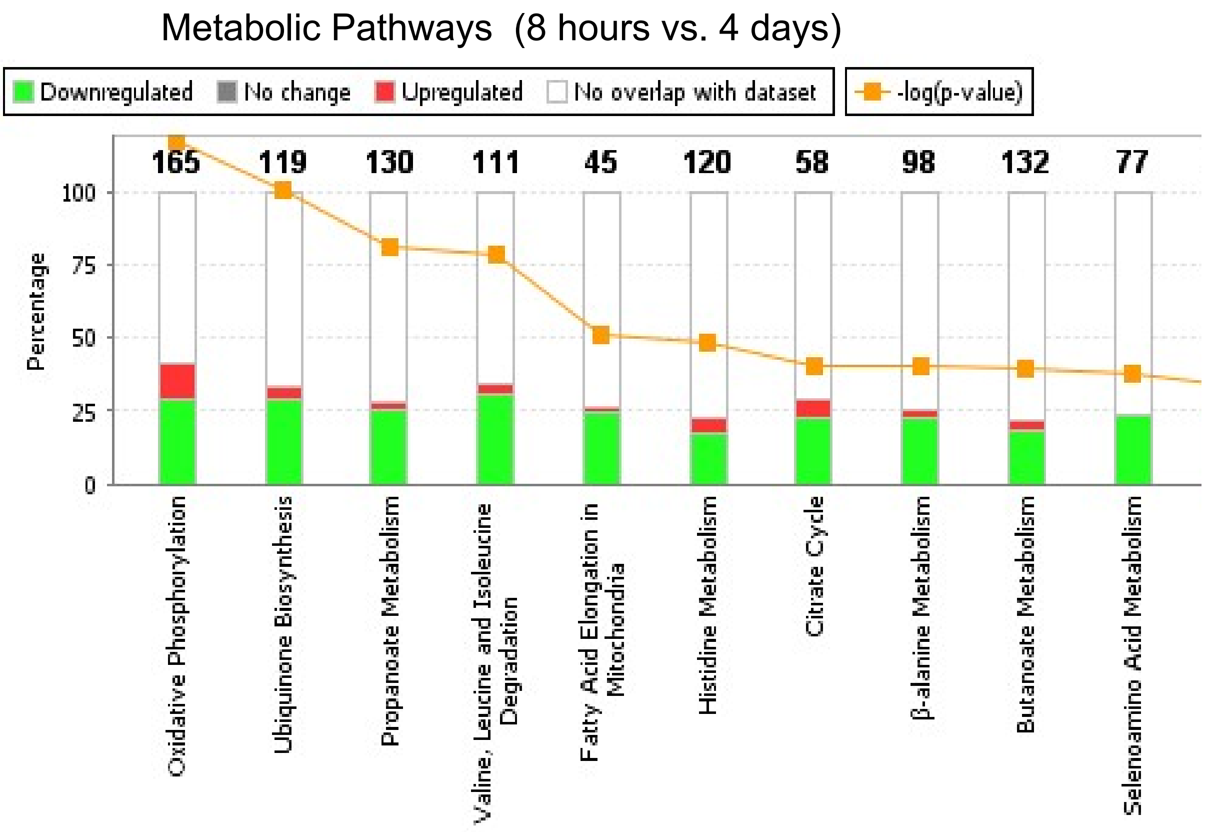

Metabolic Pathways (8 hours vs. 4 days)
Downregulated
No change
Upregulated
No overlap with dataset
-log(p-value)
Percentage
100
75
50
25
0
165
119
130
111
45
120
58
98
132
77
Oxidative Phosphorylation
Ubiquinone Biosynthesis
Propanoate Metabolism
Valine, Leucine and Isoleucine Degradation
Fatty Acid Elongation in Mitochondria
Histidine Metabolism
Citrate Cycle
β-alanine Metabolism
Butanoate Metabolism
Selenoamino Acid Metabolism

Figure S4

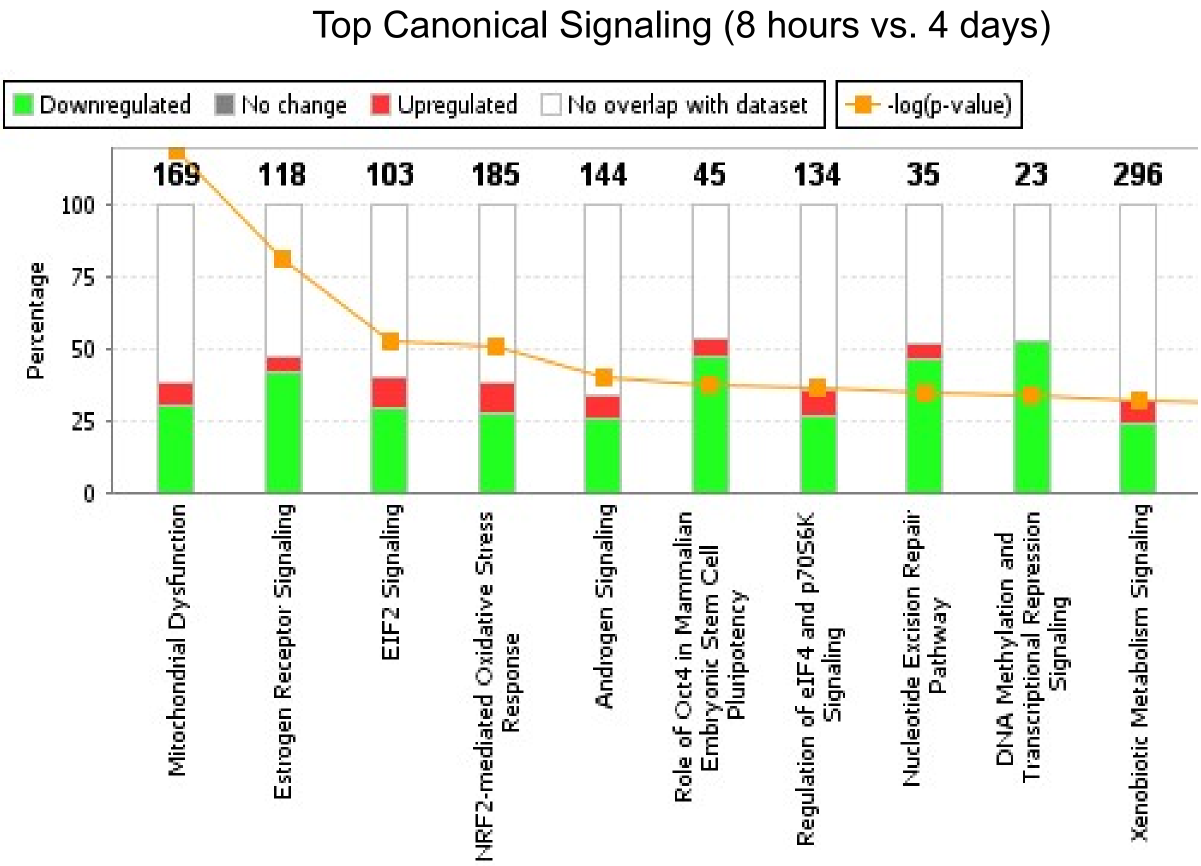

Top Canonical Signaling (8 hours vs. 4 days)
Downregulated
No change
Upregulated
No overlap with dataset
-log(p-value)
Percentage
100
75
50
25
0
169
118
103
185
144
45
134
35
23
296
Mitochondrial Dysfunction
Estrogen Receptor Signaling
EIF2 Signaling
NRF2-mediated Oxidative Stress Response
Androgen Signaling
Role of Oct4 in Mammalian Embryonic Stem Cell Pluripotency
Regulation of eIF4 and p70S6K Signaling
Nucleotide Excision Repair Pathway
DNA Methylation and Transcriptional Repression Signaling
Xenobiotic Metabolism Signaling

Figure S5

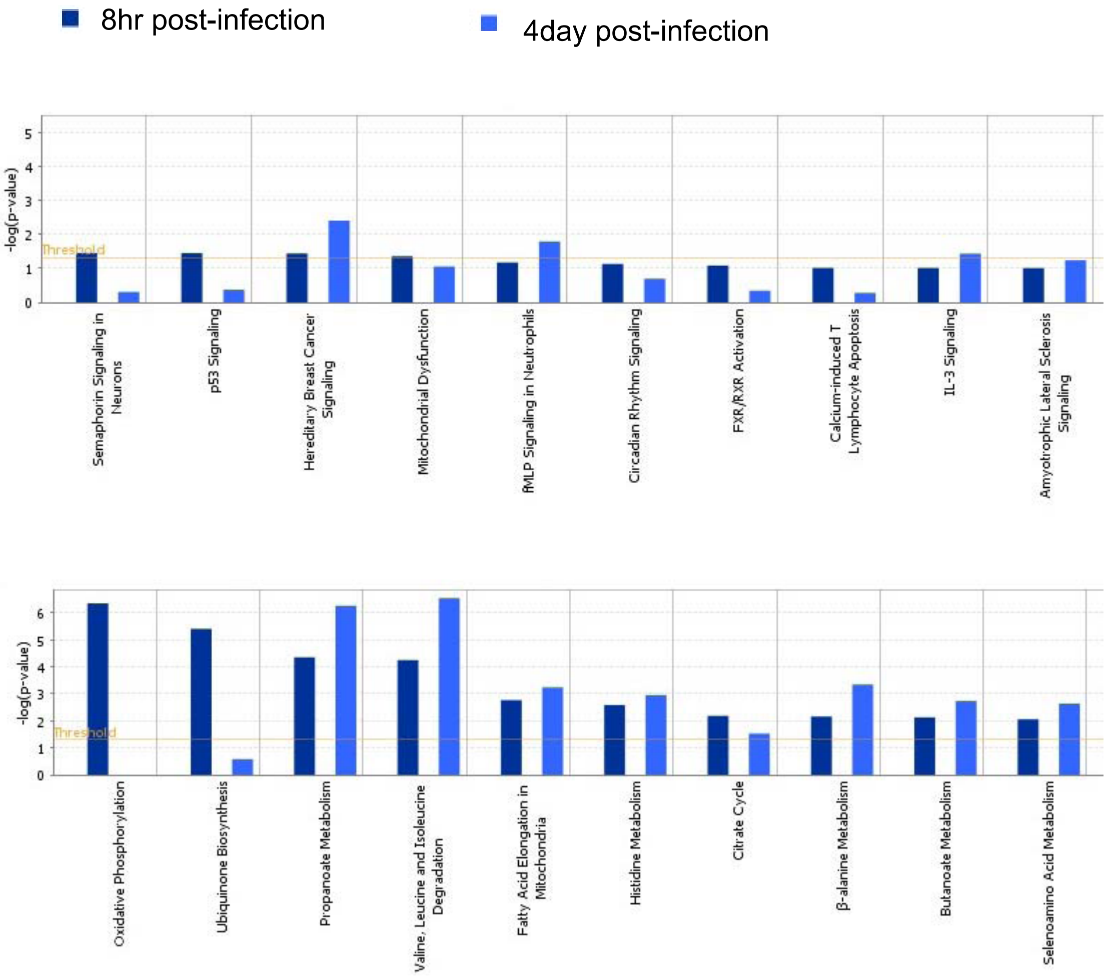
8hr post-infection
4day post-infection
-log(p-value)
Threshold
Semaphorin Signaling in Neurons
p53 Signaling
Hereditary Breast Cancer Signaling
Mitochondrial Dysfunction
fMLP Signaling in Neutrophils
Circadian Rhythm Signaling
FXR/RXR Activation
Calcium-induced T Lymphocyte Apoptosis
IL-3 Signaling
Amyotrophic Lateral Sclerosis Signaling
-log(p-value)
Threshold
Oxidative Phosphorylation
Ubiquinone Biosynthesis
Propanoate Metabolism
Valine, Leucine and Isoleucine Degradation
Fatty Acid Elongation in Mitochondria
Histidine Metabolism
Citrate Cycle
β-alanine Metabolism
Butanoate Metabolism
Selenoamino Acid Metabolism

Figure S 6

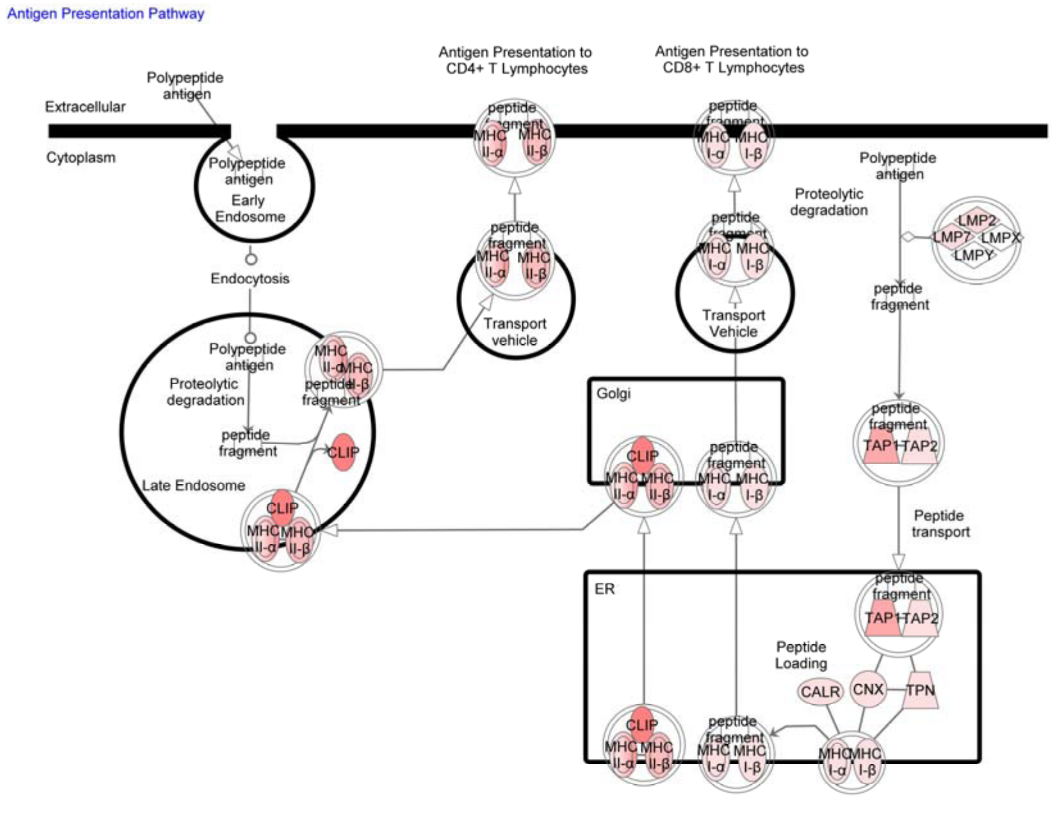

© 2000-2010 Ingenuity Systems, Inc. All rights reserved.

Figure S7

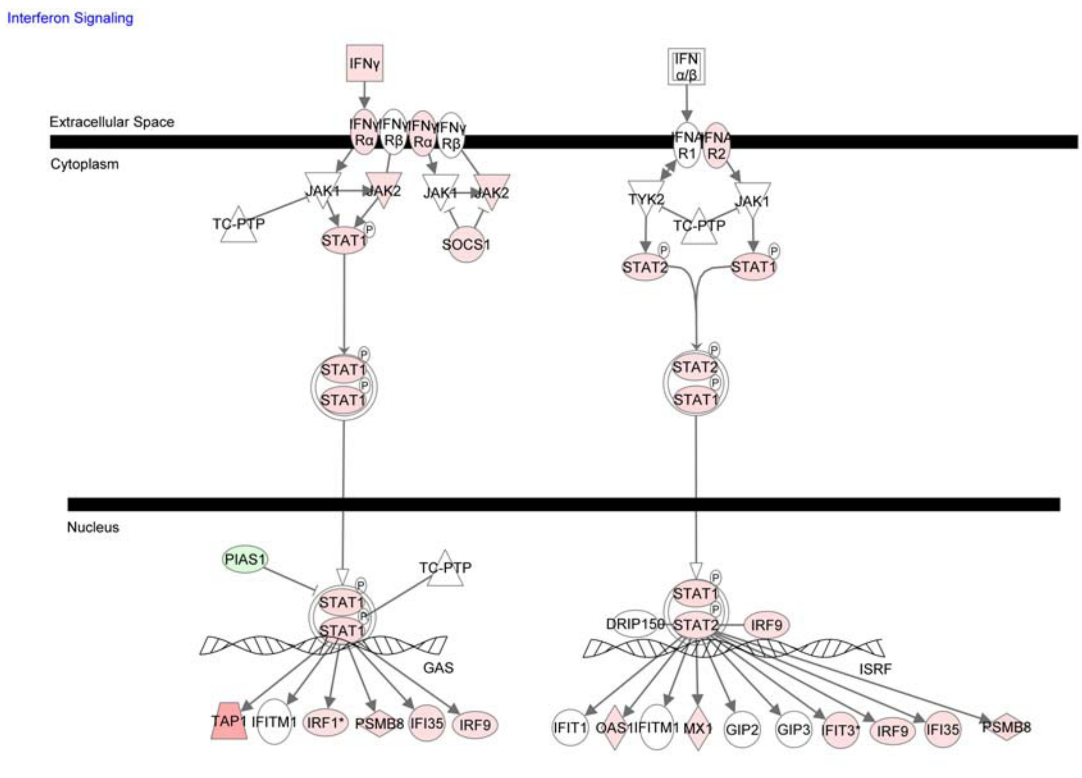

© 2000-2010 Ingenuity Systems, Inc. All rights reserved.

Figure S8

IL-4 Signaling

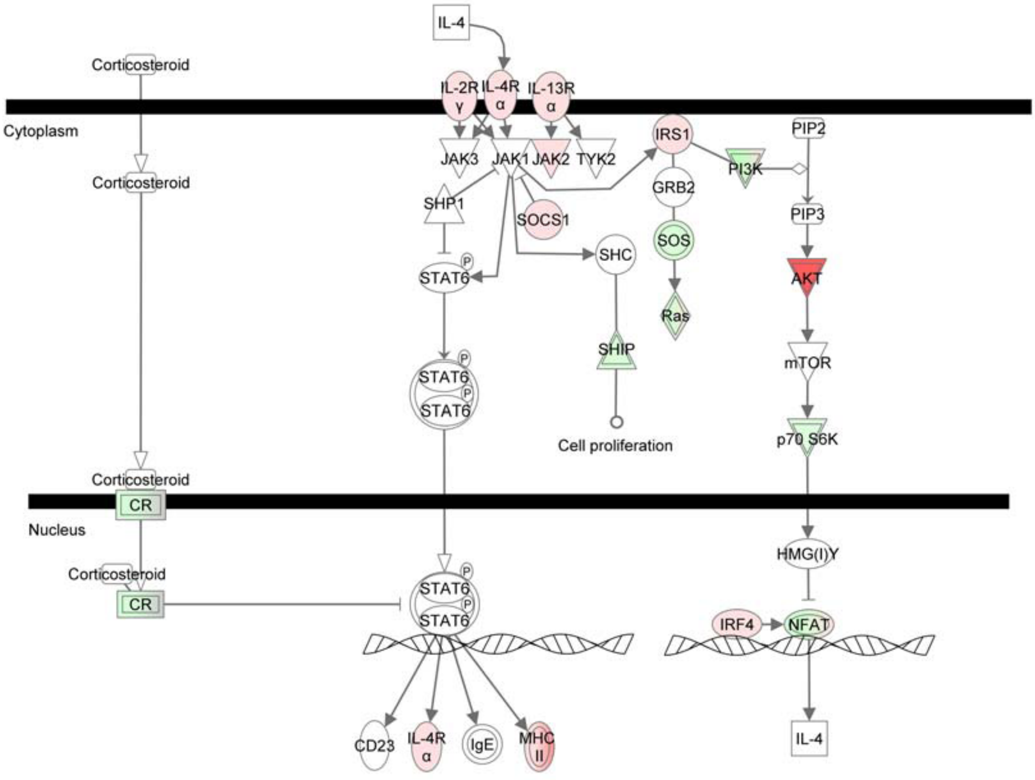

© 2000-2010 Ingenuity Systems, Inc. All rights reserved.
